# A novel PAAoptosis-inducing ERRα-targeting compound for combating hematopoietic and solid cancers

**DOI:** 10.1038/s41420-026-03010-4

**Published:** 2026-03-26

**Authors:** Wonhyoung Seo, Yerim Heo, Khang Vuong Tran, So-young Kim, Eun Jung Bae, Bokeum Jung, Sang-Hee Lee, Taylor Roh, Sang Min Jeon, Kyung Tae Kim, Eun-Jin Park, Soo In Kim, Jeong Suk Koh, Ik-Chan Song, Hyun Kyu Song, Jung-Joon Min, Jin Hee Ahn, Eun-Kyeong Jo

**Affiliations:** 1 Department of Medical Science, Chungnam National University College of Medicine, Daejeon, Republic of Korea; 2 Division of Hematology/Oncology, Department of Internal Medicine, Chungnam National University College of Medicine, Daejeon, Republic of Korea; 3 Department of Chemistry, Gwangju Institute of Science and Technology, Gwangju, Republic of Korea; 4 Department of Nuclear Medicine, Chonnam National University Medical school, Hwasun, Republic of Korea; 5 Department of Biomedical Science (BrainKorea21 Plus), Chonnam National University Graduate School, Gwangju, Republic of Korea; 6 SK Chemicals Co., Ltd., Seoul, Republic of Korea; 7 Department of Pre-medicine, Chungnam National University College of Medicine, Daejeon, Republic of Korea; 8 Center for Bio-imaging & Translational Research (104-Dong), Korea Basic Science Institute, Cheongju, South Korea; 9 Department of Chemistry, Hanyang University, Seoul, Republic of Korea; 10 Department of Life Sciences, Korea University, Seoul, Republic of Korea

**Keywords:** Chemotherapy, Preclinical research

## Abstract

Estrogen-related receptor-α (ERRα; NR3B1) is an orphan nuclear receptor that drives the progression of several cancers. To develop novel ERRα-targeting therapeutics, we designed and evaluated the function of a new compound, PAMT-001, which interacts with ERRα and effectively suppresses tumorigenesis. We demonstrated a significant interaction between ERRα and PAMT-001 using protein-small molecule binding assays and luciferase assays. Although PAMT-001 exhibited lower activity compared to the established ERRα inverse agonist XCT-790, it showed stronger anticancer effects against both hematological and solid tumors. Mechanistically, PAMT-001 promoted combined cell death mechanisms in tumors. It disrupted mitochondrial respiratory function and structure, leading to excessive production of reactive oxygen species and endoplasmic reticulum stress, ultimately resulting in apoptotic cell death. Additionally, PAMT-001 induced excessive autophagy, contributing to cancer cell death, as well as gasdermin E-mediated pyroptosis in acute myeloid leukemia and colon cancer cells. Furthermore, PAMT-001 demonstrated potential for use in precision medicine, particularly for patients with chemotherapy-resistant and NPM1-mutated acute myeloid leukemia. PAMT-001 is a potent ERRα-targeting anticancer agent capable of inducing anticancer effects through pyroptosis, autophagic cell death, and apoptosis—a newly termed mechanism referred to as “PAAoptosis.” It holds significant potential for the treatment of both hematological and solid cancers.

## Introduction

Estrogen-related receptor alpha (ERRα) is an orphan receptor that regulates mitochondrial biogenesis, energy metabolism, and redox homeostasis in major tissues and organs [1–6]. Dysregulated ERRα function is linked to metabolic disorders and also supports tumorigenic metabolic rewiring, positioning the peroxisome proliferator-activated receptor gamma coactivator 1-α (PGC1α)/ERRα axis as a key node in various disease pathogenesis [1, 3, 7–9]. Emerging evidence indicates that ERRα contributes to cancer progression, metastasis, and resistance to therapy across multiple malignancies, including breast cancer [10–14], endometrial cancer [15], ovarian cancer [16, 17], castration-resistant prostate cancer, non-small cell lung cancer [18], esophageal cancer, and acute myeloid leukemia (AML) [19]. ERRα drives tumor growth and survival through multiple mechanisms, such as favoring mitochondrial energy metabolism, mitigating reactive oxygen species (ROS) [10], maintaining cancer stemness [11], facilitating metastasis [13], and lipid metabolic reprogramming [15]. Under hypoxia, ERRα represses pyroptosis by inhibiting caspase-1/GSDMD via NLRP3 binding, promoting cisplatin resistance in endometrial cancer [20]. In EGFR inhibitor-resistant cells, ERRα re-expression drives cholesterol accumulation in lipid rafts, supporting survival [18]. These findings underscore ERRα as a key regulator of cancer cell adaptation and drug resistance.

Given its pivotal role in tumor progression and drug resistance, ERRα has emerged as a promising therapeutic target. Among its inverse agonists, XCT-790 has been widely studied for anticancer efficacy across tumor types [11, 21, 22]. XCT-790 overcomes chemoresistance of NSCLC [18] and hepatoma [23] cells, and exerts synergistic antitumor effects against pancreatic cancer [24]. It promotes apoptosis in endometrial cancer via inhibiting peroxisome proliferator-activated receptor-γ signaling [25] and suppresses androgen signaling in prostate cancer [26]. In melanoma, inhibition of the PGC1α/ERRα axis by XCT-790 or SR-18292 reduces tumorigenicity [27]. We previously demonstrated that ERRα blockade by XCT-790 or gene silencing disrupts mtOXPHOS, enhancing cytotoxicity and antileukemic activity [19]. These findings support ERRα inhibition as a compelling therapeutic strategy in oncology.

In this study, we developed a series of ERRα inhibitors based on the chemical scaffold of XCT-790 and identified PAMT-001 as a potent anticancer compound effective against both solid tumors and hematologic malignancies. Although PAMT-001 exhibited lower binding affinity to ERRα compared to XCT-790, it more effectively suppressed the growth of various cancer cells in both in vitro and in vivo models. Mechanistically, PAMT-001 disrupted mitochondrial oxidative phosphorylation (mtOXPHOS) and induced endoplasmic reticulum (ER) stress. These events collectively triggered a form of multi-modal cell death—comprising pyroptosis, apoptosis, and autophagic cell death—referred to as “PAAoptosis”. These findings position PAMT-001 as a novel therapeutic candidate with a unique mechanism of action, offering a promising strategy for treating cancers, including those with chemoresistance.

## Results

### Designing of PAMT-001 and its identification as a new inverse agonist of ERRα

We aimed to develop a new anticancer candidate compound targeting ERRα, based on the well-established potential of ERRα inhibition for cancer treatment [28–30]. We synthesized a variety of XCT-790 derivatives to develop a new ERRα modulator with therapeutic activity superior to that of XCT-790. Ether linkage in the vanillin core of XCT-790 is crucial for its interaction with ERRα [31, 32]. Based on the structure of XCT-790, 12 derivatives were designed and synthesized (details in Supplementary information). Among the synthesized compounds, PAMT-001 demonstrated significantly higher cytotoxicity compared to XCT-790 at the same concentration (Supplementary Fig. 1A).

**Fig. 1:**
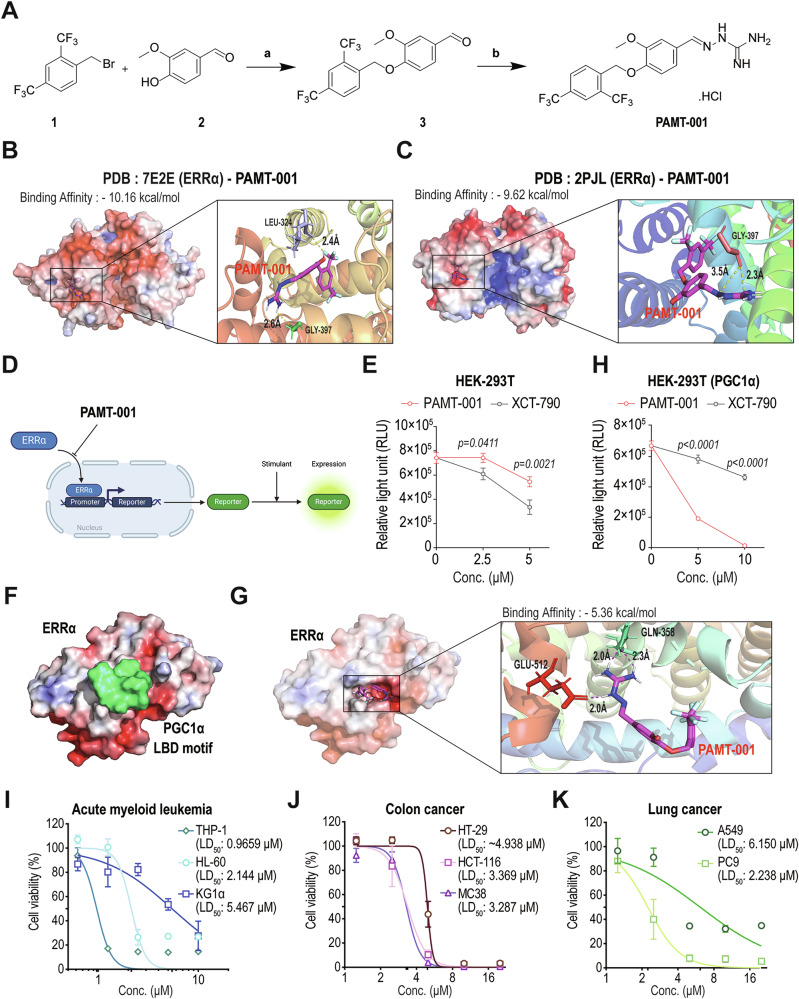
PAMT-001 inhibits ERRα activity and induces cancer cell death. **A** Synthesis of PAMT-001. Reagents and conditions: (a) K2CO3, acetone, reflux, 18 h, 89%; (b) MeOH, reflux, 18 h, 95%. Three-dimensional structures of PAMT-001 bound to ERRα at two promising druggable binding (PDB) sites (PDB: 7E2E, (**B**)) or (PDB: 2PJL, (**C**)) predicted using PyMOL. **D** Schematic diagram of the reporter gene assay. Transfection of the reporter gene plasmid containing luciferase reporter driven by ERR-alpha response element into 293T cells to confirm the inhibitory effect of PAMT-001 on ERRα based on luciferase activity. **E** Transfection of HEK293T cells with -ERRE-reporter (luciferase) plasmid followed by stimulation with XCT-790 or PAMT-001 at indicated concentrations. *P*-values were calculated using one-way ANOVA for multiple comparisons. **F**, **G** Three-dimensional structure of ERRα (PDB: 1XB7) bound to the PGC1α LBD motif (**F**). Three-dimensional structure of PAMT-001 bound to ERRα (PDB: 1XB7) demonstrating the electrostatic forces at the site of PGC1α interaction (**G**). Both structures were predicted using PylMOL. **H** Transfection of HEK293T cells with -ERRE-reporter (luciferase) plasmid for PGC1α overexpression followed by stimulation with XCT-790 or PAMT-001 at the indicated concentrations. *P*-values were calculated using one-way ANOVA for multiple comparisons. **I** CCK-8 assay of PAMT-001-treated AML cancer cell lines (THP-1, HL-60, and KG1α). *P*-values were calculated using the extra sum of squares *F*-test. MTT assay for evaluating the cytotoxicity of PAMT-001 in colon cancer (**J**) (HT-92, HCT-116, and MC38) and lung cancer (**K**) (A549 and PC-9) cell lines. LD_50_ values were calculated through [inhibitor] vs. normalized response—Variable slope in Prism 8 (**I**–**K**). Data represent means ± SD from more than three independent experiments (**E**, **H**, **I**–**K**).

The synthesis of PAMT-001 is illustrated in Fig. 1A. A commercially available compound 1 was reacted with vanillin (compound 2) in the presence of a base, yielding intermediate 3. Subsequently, aldehyde 3 was coupled with aminoguanidine hydrochloride under reflux conditions to produce the final compound, PAMT-001. Computational studies using MolModa showed that PAMT-001 effectively binds to the ERRα ligand-binding domain with a high affinity score in different models (Fig. 1B, C), (supplementary Fig. 1B, C). However, PAMT-001 showed a weaker EC_50_ value (0.17 µM) compared to XCT-790 (0.038 µM) in ERRα activity assays (Supplementary Fig. 1D), corresponding to lower inhibitory efficiency in the reporter gene assay of ERRα (Fig. 1E). Because PAMT-001 alone exhibited limited inhibitory effects on ERRα, we explored alternative mechanisms for regulating ERRα function, focusing on cofactors of ERRα, like PGC1α. In silico modeling with PyMOL was used to investigate whether PAMT-001 could bind to the PGC1α-binding site on ERRα. PGC1α is a critical coactivator of ERRα that enhances ERRα‘s transcriptional activation of mitochondrial metabolism [33, 34] (Fig. 1F). The computational analysis suggested that PAMT-001 binds to the same ERRα location as PGC1α (Fig. 1G). Moreover, reporter gene assay demonstrated that PAMT-001 suppressed ERRα activity more significantly than XCT-790 when PGC1α was overexpressed (Fig. 1H). These findings indicated that PAMT-001 interacts with and inhibits ERRα through cooperation with PGC1α.

As cytotoxicity against KG1α cells (Supplementary Fig. 1A), PAMT-001 was potently cytotoxic against various cancer cell lines, although lethal dose 50% (LD_50_) values varied among cell types (Fig. 1I–K). The microscope confirmed fragmented organelles and disrupted membrane integrity in HL-60 cells after exposure to PAMT-001 (Supplementary Fig. 1E). Thus, we chose PAMT-001, a novel ERRα-targeting agent, for further experiments.

### PAMT-001 inhibits mtOXPHOS and impairs mitochondrial respiration in various cancer cells

**Fig. 2:**
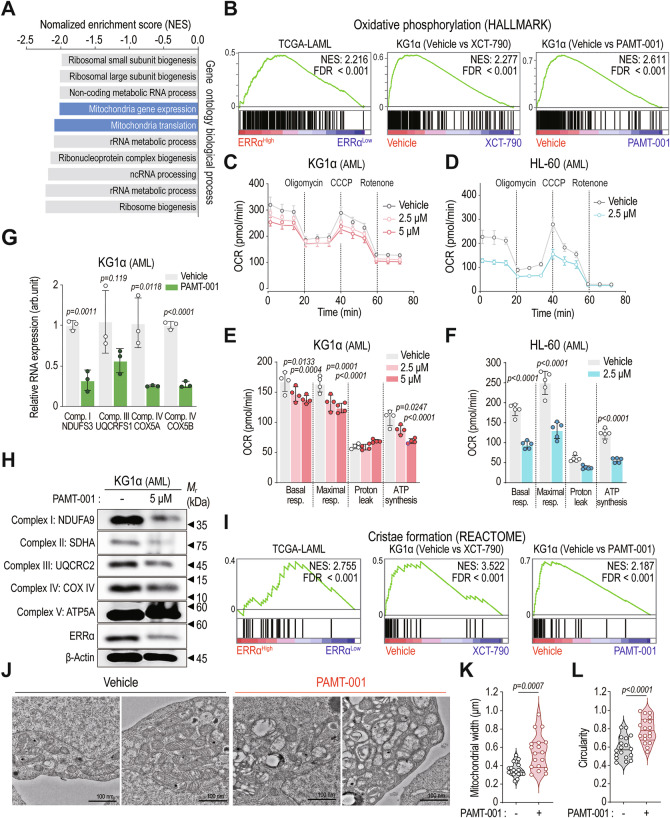
PAMT-001 inhibits mitochondrial respiration and distorts mitochondrial structure. **A** Normalized enrichment score (NES) plot of the bottom 10 pathways via gene set enrichment analysis (GSEA) of PAMT-001-treated KG1α cells. **B** GSEA against Hallmark: oxidative phosphorylation. Genes were ranked based on fold changes between ERRα-high and -low (left), vehicle and XCT-790 (middle), and vehicle and PAMT-001 (right). Oxygen consumption rate (OCR) decreased with PAMT-001 treatment in KG1α (**C**) and HL-60 (**D**) cells. For each time point, mean ± SD value of OCR evaluated via Seahorse XF analysis is presented (*n* = 4 for KG1α; *n* = 5 for HL-60). Summary of OCR for mitochondrial respiration-related markers (basal and maximal respiration, proton leakage, and ATP production) of KG1α (**E**) and HL-60 (**F**) cells. *P*-values were calculated using one-way ANOVA (**E**) and two-tailed *t*-test (**F**). **G** Quantification of the expression of OXPHOS genes (*NDUFS3*, *UQCRFS1*, *COX5A*, and *COX5B*) using qRT-PCR in PAMT-001-treated KG1α cells (5 μM, 12 h). *P*-values were calculated using a two-tailed *t*-test (mean ± SD, *n* = 3). **H** Protein levels of OXHPOS complexes (NDUFA9, SDHA, UQCRC2, COX IV, and ATP5A) in response to PAMT-001 treatment (5 μM, 12 h) in KG1α cells. **I** GSEA against Reactome: cristae formation. Genes were ranked based on fold changes between ERRα-high and -low (left), vehicle and XCT-790 (middle), and vehicle and PAMT-001 (right). **J** Representative transmission electron microscopy images of vehicle-treated- or PAMT-001-treated HL-60 cells. Mitochondria of PAMT-001-treated cells showed mitochondrial damage, such as the disappearance and widening of cristae. Quantification of the mitochondrial width (**K**) and circularity (**L**) in 18 randomly selected mitochondria from five cells (**J**). *P*-values were calculated using a two-tailed *t*-test (mean ± SD, *n* = 18).

To investigate the mechanism of action of PAMT-001 in cancer cells, gene set enrichment analysis (GSEA) was performed on RNA-seq data from PAMT-001-treated KG1α cells (Fig. 2A). GSEA showed a significant downregulation of gene signature associated with mitochondrial function, as derived from Gene Ontology Biological Processes (GOBP; Fig. 2A). Furthermore, consistent with the strong correlation between ERRα expression and mOXPHOS gene expression [19] (Fig. 2B, left), we confirmed that both XCT-790 and PAMT-001 markedly decreased mtOXPHOS gene expression in KG1α cells (Fig. 2B, middle and right), underscoring their metabolic impact.

Mitochondrial respiration, assessed via Seahorse assay, demonstrated a significant decrease in oxygen consumption rate (OCR) in both KG1α (Fig. 2C) and HL-60 cells (Fig. 2D), following PAMT-001 treatment. This reduction was reflected in decreased basal respiration, maximal respiration, and ATP production, indicating mitochondrial dysfunction by PAMT-001 (Fig. 2E, F). Notably, PAMT-001 reduced the mRNA levels of key OXPHOS genes, including *NDUFS3* (complex I), *UQCRFS1* (complex III), and *COX5A/5B* (complex IV) (Fig. 2G), along with reduced protein expression of NDUFA9 (complex I), UQCRC2 (complex III), and COX IV (complex IV) (Fig. 2H). Similar decreases were observed in HL-60 and HCT-116 cells (Supplementary Fig. 2A, B), paralleling the effects of XCT-790 (Supplementary Fig. 2C), supporting ERRα inhibition as the underlying mechanism [28, 35].

Given the strong influence on mitochondrial respiration (Fig. 2A), we examined mitochondrial structure. GSEA revealed a positive correlation between ERRα expression and genes involved in mitochondrial cristae formation in the TCGA-LAML cohort (Fig. 2I, left). Treatments of XCT-790 and PAMT-001 showed gene expression profiles resembling those observed with patients exhibiting low ERRα expression (Fig. 2I, middle and right). In PAMT-001-treated HL-60 cells, transmission electron microscopy (TEM) showed increased mitochondrial width, circularity, and swelling (Fig. 2J–L). HCT-116 cells treated with PAMT-001 also exhibited distorted inner mitochondrial membrane cristae (Supplementary Fig. 2D), indicative of mitochondrial dysfunction [36]. Overall, these findings demonstrate that PAMT-001 disrupts mitochondrial function by downregulating mtOXPHOS genes and compromising mitochondrial structure.

### PAMT-001 exhibits cytotoxic effects in various cancers through mitochondria-mediated apoptosis

**Fig. 3:**
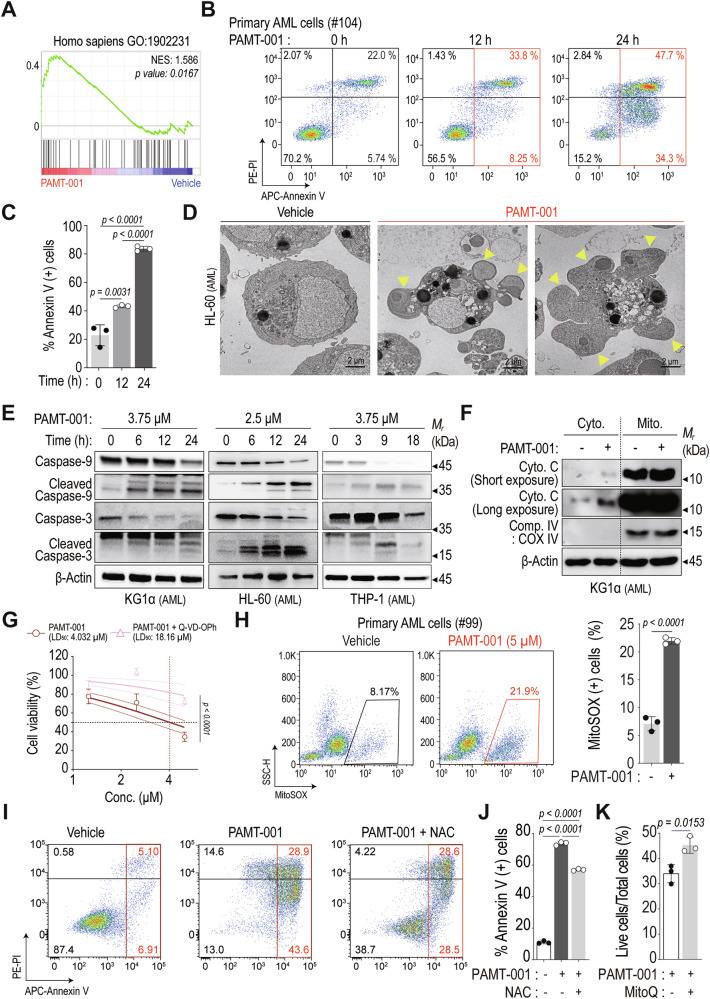
PAMT-001-mediated mitochondrial dysfunction and mtROS activate the intrinsic apoptotic process. **A** GSEA against GOBP_Positive regulation of intrinsic apoptosis. Genes were ranked based on fold changes between vehicle and PAMT-001. **B**, **C** Flow cytometry showing apoptosis of Annexin V/PI-stained primary AML cells #104 (**B**). Bar plot showing fractions (%) of Annexin V-positive cells (**C**). *P*-values were calculated using one-way ANOVA for multiple comparisons (mean ± SD, *n* = 3). Representative transmission electron microscopy image of apoptotic blebbing (yellow arrowhead) in PAMT-001-treated HL-60 cells (2.5 µM, 12 h). **E** Western blotting of apoptosis-associated proteins in PAMT-001 treatment. Time-dependent cleavage of caspase-9 and -3 by PAMT-001 in AML cell lines (KG1α (left), HL-60 (middle), and THP-1 (right). **F** Western blotting of cytochrome C released from mitochondria into the cytoplasm in PAMT-001-treated KG1α cells. **G** LD_50_ value of PAMT-001 with and without Q-VD-OPh in KG1α analyzed using MTT assay. LD_50_ values were calculated through [inhibitor] vs. normalized response—Variable slope in Prism 8. *P*-values were calculated using the extra sum of squares *F*-test (mean ± SD, *n* = 4). **H** Representative flow cytometry of mtROS levels in primary AML cells (#99) treated with PAMT-001 (5 μM, 18 h), as detected via MitoSOX staining. The bar plot shows fractions (%) of MitoSOX-positive cells. *P*-values were calculated using a two-tailed *t*-test (mean ± SD, *n* = 3). **I**, **J** Cell death measured using flow cytometry with Annexin V/PI staining of KG1α cells. Cells were cotreated with N-acetylcysteine (NAC) and PAMT-001 (3.75 μM) for 18 h (**I**). Bar plot shows fractions (%) of Annexin V-positive cells (**J**). *P*-values were calculated using one-way ANOVA for multiple comparisons (mean ± SD, *n* = 3). **K** Cell death was measured using the tryptophan exclusion assay of K562-luc cells. PAMT-001-induced cell death was partially rescued by MitoQ. *P*-values were calculated using a two-tailed *t*-test (mean ± SD, *n* = 3).

Similar to XCT-790 [19, 37], PAMT-001 treatment enriched apoptosis-associated gene sets in GOBP in KG1α cells (Fig. 3A). PAMT-001 increased annexin V-positive populations in HL-60 (Supplementary Fig. 3A, B) and primary AML cells (Fig. 3B, C). TEM images of HL-60 cells showed membrane blebbing, a hallmark of apoptosis (Fig. 3D). Transcriptomic data suggested the activation of the intrinsic apoptotic pathway (Fig. 3A), supported by the cleavage of caspase-9 and caspase-3 observed in AML cell lines (Fig. 3E) and HCT-116 cells (Supplementary Fig. 2C). PAMT-001 further induced mitochondrial fission in KG1α cells (Supplementary Fig. 2D), consistent with features of intrinsic apoptosis [38, 39], and triggered cytochrome c release into the cytosol (Fig. 2F). To confirm the role of caspase-dependent apoptosis, co-treatment with the pan-caspase inhibitor Q-VD-OPh [40, 41] led to a threefold increase in the LD_50_ of PAMT-001 in KG1α cells (Fig. 3G). Another pan-caspase inhibitor (Z-VAD-FMK) rescued cell viability in PAMT-001-treated HL-60 (Supplementary Fig. 3E) and KG1α (Supplementary Fig. 3F) cells. Q-VD-OPh also significantly raised the LD_50_ of PAMT-001 in solid cancer cell lines such as HCT-116 (Supplementary Fig. 3G) and A549 (Supplementary Fig. 2H) cells.

Given that mitochondrial dysfunction generates mtROS [42], particularly at complex I and III [43–45], and PAMT-001 downregulated these complexes (Fig. 2G, H, Supplementary Fig. 2A, B), we assessed mtROS levels. MitoSOX staining revealed increased mtROS in primary AML and KG1α cells (Fig. 3H, Supplementary Fig. 3I, J). ROS scavengers, including N-acetylcysteine (NAC) and MitoQ, partially but significantly rescued PAMT-001-induced cell death in various cancer cells (Fig. 3I–K, Supplementary Fig. 3K, L), suggesting that mtROS contributes to apoptosis. These results highlight the multifaceted nature of PAMT-001-induced cytotoxicity involving mtROS-mediated apoptotic signaling.

### ER stress, particularly CHOP, contributes to PAMT-001-induced cell death

**Fig. 4:**
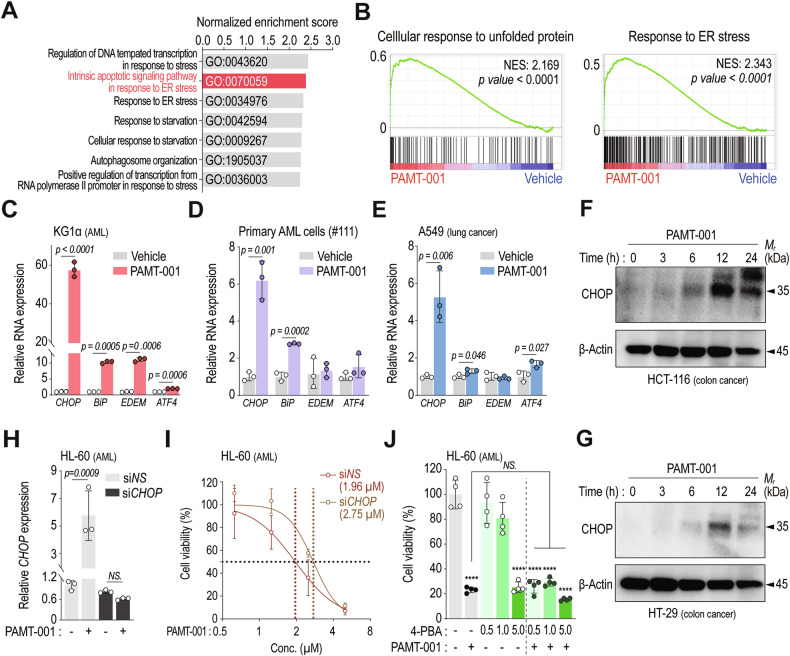
PAMT-001 promotes cell death by stimulating ER-stress-related genes, especially CHOP. **A**, **B** GSEA against GOBP: Cellular response to unfolded protein (**B**, left) and GOBP: Response to endoplasmic reticulum stress Genes (**B**, right) were ranked based on fold changes between vehicle and PAMT-001 treated KG1α cells. Quantification of gene expression of ER stress-related gene (*CHOP, BiP, EDEM*, and *ATF4*) was measured by qRT-PCR at the indicated concentration and time in KG1α (**C**), primary AML cell (**D**), and A549 (**E**). Of these, PAMT-001 primarily increased *CHOP* expression. *P-values* were calculated by a two-sided *t-test* (mean ± SD, *n* = 3, respectively). The western blot analysis of PAMT-001-induced CHOPA expression in HCT-116 (**F**) and HT-29 (**G**). **H** Quantification of *CHOP* expression induced by PAMT-001 (2.5 μM, 24 h) in HL-60 with transfection of siRNA targeting CHOP (si*CHOP*) and non-targeting (si*NS*). *P-values* were calculated by one-way ANOVA for multiple comparisons (mean ± SD, *n* = 3, respectively). **I** The LD_50_ value of PAMT-001 according to *CHOP* expression in HL-60 cells. The LD_50_ values were calculated through [inhibitor] vs. normalized response—Variable slope in Prism 8. *P-values* were calculated by the extra sum of squares *F*-test. **J** The cytotoxicity analysis for detecting rescues PAMT-001-induced cell death by ER stress inhibitor (4-PBA; 4-Phenylbutyric acid) using CCK-8 assay in HL-60. *P-values* were calculated by one-way ANOVA for multiple comparisons (mean ± SD). ****P < *0.0001*.

The ER works closely in association with mitochondria to maintain cellular homeostasis and aids in determining cell fate [46, 47]. Mitochondria-derived oxidative stress disrupts the redox state of the ER, impairing disulfide bond formation and protein folding, which leads to ER stress [48]. Based on this, we hypothesized that mitochondrial dysfunction triggers ER stress, contributing to cell death. GSEA showed enrichment of ER stress-related gene sets in PAMT-001-treated KG1α cells (Fig. 4A, B). qRT-PCR confirmed upregulation of ER stress markers *BiP*, *EDEM*, *ATF4*, and especially *CHOP* in multiple cancer cell types (Fig. 4C–E). CHOP protein levels also increased over time after PAMT-001 treatment (Fig. 4F, G, Supplementary Fig. 4A, B). As CHOP mediates ER stress-induced apoptosis [49], we used siRNA to assess its role in PAMT-001-induced cytotoxicity. CHOP knockdown modestly improved viability in 293T cells (Supplementary Fig. 4B, C) and significantly increased resistance to PAMT-001 in HL-60 cells, as shown by a higher LD_50_ (Fig. 4H, I). However, treatment with the ER stress inhibitor 4-phenylbutyric acid (4-PBA) did not prevent cell death (Fig. 4J). This indicated that although PAMT-001 induced ER stress, this stress itself was not the primary cause of cell death. Instead, it played a contributory role, particularly via the upregulation of CHOP.

### PAMT-001 triggers autophagy for CHOP signaling and apoptosis

**Fig. 5:**
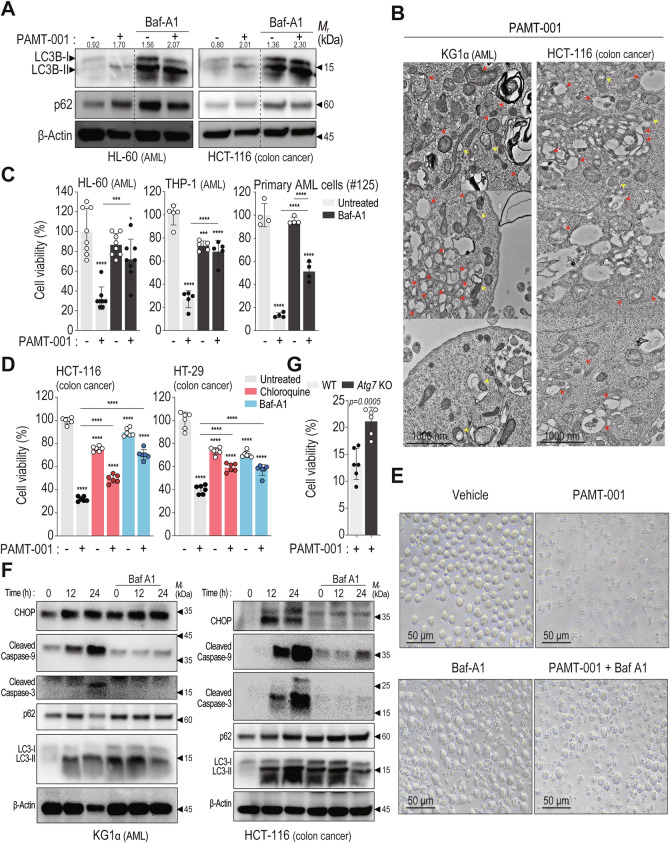
PAMT-001-mediated autophagy contributes to apoptotic cell death. **A** Western blotting for PAMT-001-induced autophagic flux in HL-60 (left) and HCT-116 (right) cells. **B** Representative TEM images of PAMT-001-treated KG1α (left) and HCT-116 (right) cells. Multiple double-membrane autophagosomes (red arrow) or mitophagosomes (yellow arrow) accumulated in the cytoplasm. **C**, **D** Cytotoxicity analysis for detecting the rescue of PAMT-001-induced cell death by autophagy inhibitor (bafilomycin A1 and chloroquine) using trypan blue exclusion assay for HL-60 cells, CCK-8 assay for THP-1 cells, and MTT assay for primary AML #125, HCT-116, and HT-29 cells. Cells were cotreated with PAMT-001 and bafilomycin or chloroquine for 24 h (HL-60 and THP-1), 48 h (primary AML cell #125), or 30 h (HCT-116 and HT-29). *P*-values were calculated using one-way ANOVA for multiple comparisons (mean ± SD). **E** Optical microscopy images showing PAMT-001-induced HL-60 cell death and its rescue by bafilomycin A1. **F** Western blotting for autophagy-dependent cell death in KG1α (left) and HCT-116 cells(right). Expression of apoptotic markers (cleaved caspase-3 and caspase-9) and CHOP was attenuated by bafilomycin A1 treatment. **G** MTT assay for detecting ATG7-dependent cell death. HT-29 with ATG7 knockout (KO) cells showed more resistance to PAMT-001-induced cell death than those with ATG7 wild type. *P*-values were calculated using a two-sided *t*-test (mean ± SD, *n* = 6). ***P < 0.001, ****P < 0.0001.

PAMT-001-treated KG1α cells were enriched in gene sets related to autophagy as well as ER stress (Fig. 4A). Notably, PAMT-001 strongly induced autophagy flux in various cell lines (Fig. 5A, Supplementary Fig. 5A). In HL-60 and HCT-116 cells, the LC3 II/I ratio increased following treatment with PAMT-001 and the lysosomal acidification inhibitor bafilomycin A1 [50], indicating enhanced autophagic flux (Fig. 5A). TEM analysis revealed abundant double-membrane vesicles in PAMT-001-treated KG1α and HCT-116 cells (Fig. 5B, red/yellow arrow), confirming autophagy activation.

Autophagy in cancer plays a double-edged role [51, 52], supporting both cancer survival and death in the context-dependent [53]. In our study, the treatment of PAMT-001 with autophagy inhibitors, bafilomycin A1 or chloroquine, significantly rescued cell viability in AML, primary AML, and colon cancer cells (Fig. 5C, D). In HL-60 cells, bafilomycin A1 visibly reduced PAMT-001-induced cytotoxic effects (Fig. 5E). Additionally, autophagy inhibition reduced CHOP expression and caspase-3/9 cleavage (Fig. 5F, Supplementary Fig. 5B), indicating that autophagy may function upstream of apoptosis and ER stress. Knocking out *ATG7*, a key regulator of autophagy, failed to completely prevent cell death (Fig. 5G). These findings suggest that PAMT-001 induces autophagy, which plays a critical—though not exclusive—upstream role in CHOP signaling and apoptosis during PAMT-001-induced cancer cell death.

### PAMT-001 induces pyroptotic cell death via GSDME cleavage

**Fig. 6:**
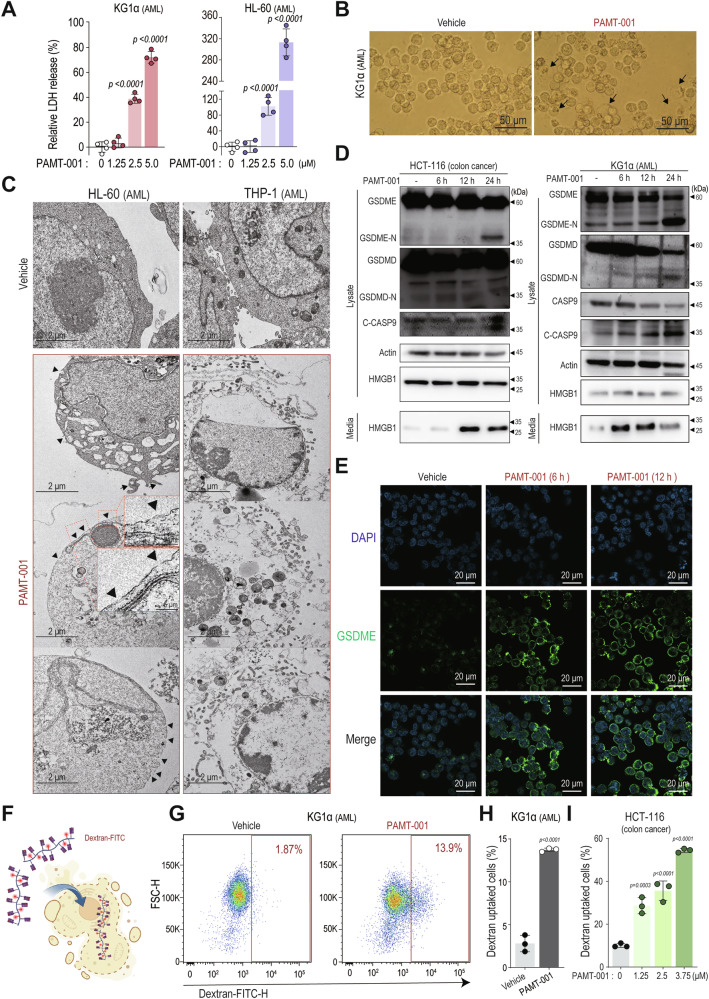
PAMT-001 induces the cleavage of GSDME and executes pyroptosis. **A** Effect of PAMT-001 on LDH release from KG1α (left) and HL-60 cells (right). *P-values* were calculated by one-way ANOVA for multiple comparisons (mean ± SD). **B** Optical microscope images showing PAMT-001-induced cell death with bubble-like protrusion (black arrow) in KG1α cells. **C** Representative TEM images of vehicle-treated- or PAMT-001-treated HL-60 (left) and THP-1 cells(right). The cell surfaces of PAMT-001-treated HL-60 lack continuity with membrane pores (black arrowheads) and cytoplasmic protrusions (black arrow). In PAMT-001-treated THP-1, the intracellular organelles are damaged and leak into the extracellular spaces as a result of the disrupted plasma membrane. **D** Western blot analysis for pyroptotic cell death in HCT-116 (left) and KG1α cells (right). The cleavage of GSMDE and the release of HMGB1 from the cytosol to the extracellular space in PAMT-001-treated HCT-116 and KG1α. **E** Representative images of GSDME-stained KG1α cells (green) were obtained by confocal microscopy. PAMT-001 (3.75 μM) was treated for 12 h. **F** A schematic diagram of the experiments for proving the membrane pore using dextran-FITC. **G**–**I** The flow cytometry measuring the entrance of dextran into the intracellular space through membrane pores in KG1α (**G**). The bar plot showing fractions (%) of dextran contained KG1α (mean ± SD, *n* = 3, respectively) (**H**) and HCT-116 (mean ± SD, *n* = 3, respectively) (**I**). *P-value* was calculated by two-sided *t-*test (**H**) or one-way ANOVA for multiple comparisons (**I**).

Recent studies show that caspase-3 can trigger pyroptosis by cleaving gasdermin E (GSDME) [54–56]. To assess whether PAMT-001 induces pyroptosis, we measured LDH release as a marker of membrane damage. A dose-dependent increase in LDH release was observed in KG1α, HL-60 (Fig. 6A), and HCT-116 cells (Supplementary Fig. 6A). KG1α cells displayed typical pyroptotic morphology, including cell rounding and membrane blebbing (Fig. 6B). TEM analysis confirmed the formation of pyroptotic pores and membrane rupture with bubble-like protrusions (Fig. 6C, Supplementary Fig. 6B) [57, 58]. Western blotting revealed PAMT-001-induced cleavage of GSDME into its active N-terminal fragment in all tested cancer cells, while GSDMD cleavage occurred only in KG1α cells (Fig. 6D). PAMT-001 also triggered the release of the pyroptosis marker high mobility group box 1 (HMGB1). Confocal microscopy showed increased GSDME expression and its translocation to the plasma membrane in PAMT-001-treated KG1α cells (Fig. 6E). Flow cytometry with dextran-FITC further confirmed that PAMT-001 generated cell membrane holes (Fig. 6F). PAMT-001 treatment enhanced dextran translocation into KG1α and HCT-116 cells by creating membrane pores, resulting in a higher proportion of dextran-positive cells (Fig. 6G–I). These results suggest that PAMT-001 induces GSDME-mediated pyroptosis, contributing to cancer cell death alongside apoptosis and autophagy, thus highlighting its potential as a multi-modal anticancer agent.

### In vivo anticancer effects and clinical implications of PAMT-001

**Fig. 7:**
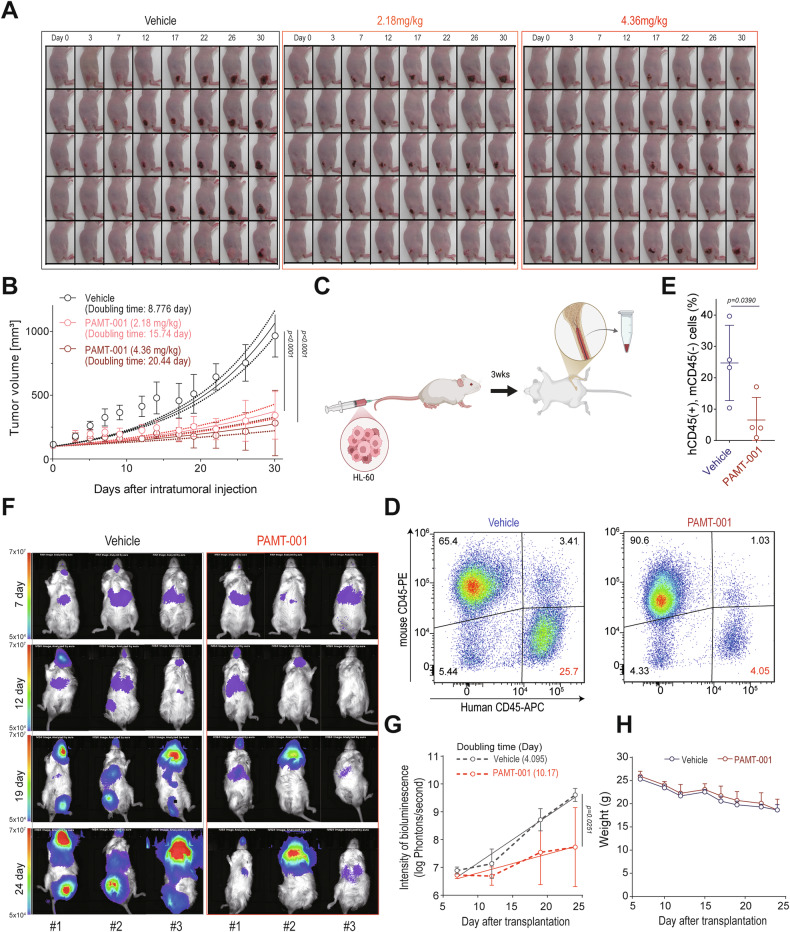
Anti-cancer effect of PAMT-001 in vivo mouse models. **A**, **B** Subcutaneous growth of HCT-116 cells treated with PAMT-001 (2.18 or 4.36 mg/kg) or vehicle. Tumor volumes were measured with a caliper twice a week. Data are presented as mean ± SD for each day. *P*-values were calculated using the extra sum of squares *F*-test. **C** Schematic diagram of the experiments performed using an orthotropic animal model of AML. **D** Representative flow cytometry plots showing the engraftment of HL-60 cells with or without PAMT-001 treatment at 4 weeks post-transplantation (left). Irradiated NOD/SCID mice were injected with HL-60 cells (5 × 10^6^ cells per mouse). **E** Ratio of HL-60 cells (human CD45+ (hCD45+) and murine CD45− (mCD45−)) in the bone marrow. *P*-values were determined using a two-sided *t*-test (mean ± SD, *n* = 4). **F** NOD/SCID/Il2rg null mice were orthotopically implanted with K562-luc cells (5 × 10^6^ cells/mouse) and administered either a vehicle (PBS) or PAMT-001 (6 mg/kg) seven days post-confirmation of K562-luc cell engraftment. Tumor proliferation was assessed via bioluminescence using the IVIS 200 system. The specified dates denote “days” post-transplantation. **G** Measurement of luminescent intensity of photons emitted from each tumor in the images presented in (**F**). *P*-values were determined using the extra sum of squares *F*-test. **H** Change in body weight of NOD/SCID/Il2rg null mice implanted with K562-luc cells upon vehicle or PAMT-001 administration.

**Fig. 8:**
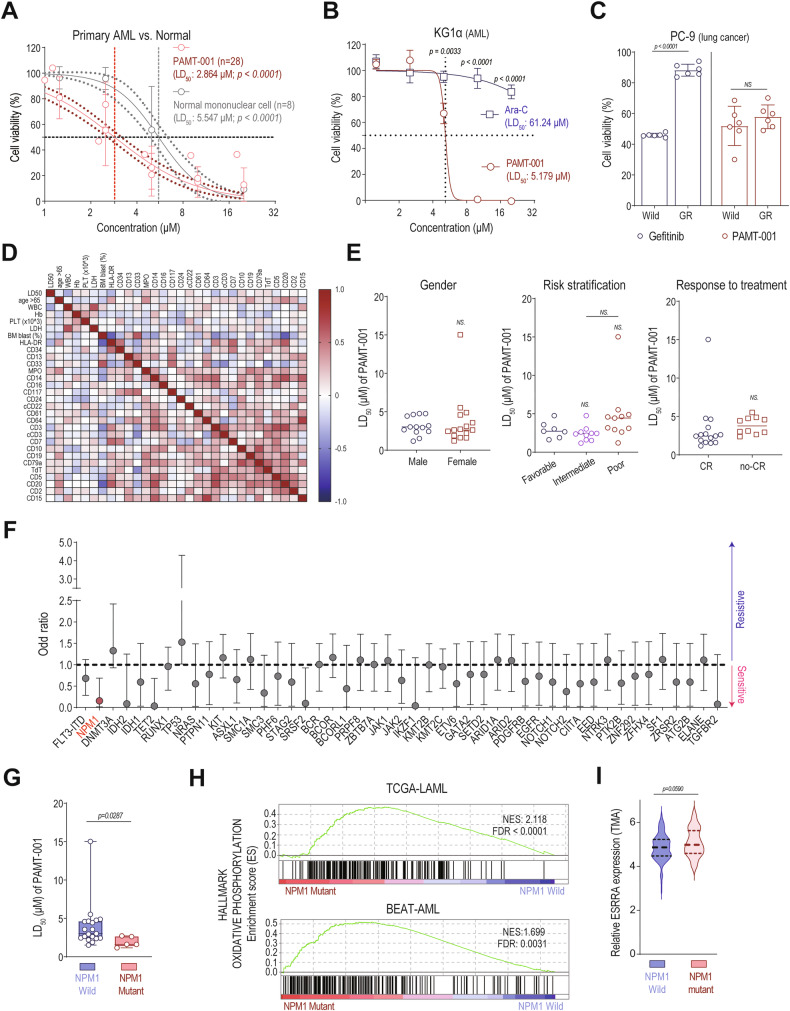
Clinical implications of PAMT-001 in cancers with treatment resistance and AML patients, particularly those with NPM1 mutations. **A** CCK-8 assay of PAMT-001-treated primary AML (*n* = 28) and normal mononuclear (*n* = 8) cells. LD_50_ values were calculated through [inhibitor] vs. normalized response—Variable slope in Prism 8. *P*-values were calculated using the extra sum of squares *F*-test (mean ± SD). **B** CCK-8 assay of PAMT-001- or cytarabine-treated KG1α cells. LD_50_ values were calculated through [inhibitor] vs. normalized response—Variable slope in Prism 8. *P*-values were calculated using a two-sided *t*-test (mean ± SD, *n* = 4). **C** MTT assay of gefitinib- or PAMT-001-treated PC-9 cells (lung cancer). *P*-values were calculated using a two-sided *t*-test (mean ± SD, *n* = 6). **D** Correlation analysis of LD_50_ value for PAMT-001 and AML patient characteristics using Pearson correlation coefficients. **E** LD_50_ value for PAMT-001 according to the gender of the patient with AML (left), risk stratification based on NCCN guidelines (middle), and responses against standard treatment (Idarubicin and cytarabine) (right). *P*-value was calculated using a two-sided *t-*test (gender and response against standard treatments) or one-way ANOVA for multiple comparisons (risk stratification). **F** Simple logistic regression between the mutation profiles of patients with AML and LD_50_ of PAMT-001. **G** Primary AML cells with NPM1 mutation showed more sensitivity to PAMT-001 than cells with normal NPM1 status. *P*-value was calculated using a two-sided *t*-test (mean ± SD). **H** Gene set enrichment analysis against Hallmark oxidative phosphorylation. Genes were ranked based on fold changes between normal and mutant NPM1 status in TCGA-LAML (top) and BEAT-AML (bottom) cohorts. **I** Relative ESRRA expression according to NPM1 status in the TCGAL-LAML cohort. *P*-value was calculated using a two-sided *t*-test (mean ± SD). CR complete remission.

We evaluated the anticancer effects of PAMT-001 in vivo using multiple animal models. In a colorectal cancer xenograft model, intratumoral injection of PAMT-001 significantly suppressed tumor growth. Treated mice showed a 2–3-fold increase in tumor doubling time compared to vehicle controls (vehicle: 13.28 days vs. 2.18 mg PAMT-001/kg: 27.95 days, vs. 4.36 mg PAMT-001/kg: 35.27 days; *p* < 0.0001) (Fig. 7A, B). In an AML mouse model, PAMT-001 treatment reduced human CD45^+^ HL-60 cells in the bone marrow, as shown by flow cytometry (Fig. 7C–E). Furthermore, in an orthotopic AML model assessed by bioluminescence imaging, PAMT-001 lowered tumor burden and slowed growth, with evidence of tumor regression in one mouse (Fig. 7F, G). Additionally, PAMT-001 exhibits promising therapeutic properties against cancer, with no apparent detrimental effects on normal cells or animals (Fig. 7H). Furthermore, AML patient-derived cells were more sensitive to PAMT-001 (LD_50_: 2.856 µM) compared to normal mononuclear cells (LD_50_: 5.158 µM, Fig. 8A). The drug effectively targeted cytarabine-resistant KG1a cells (Fig. 8B) and gefitinib-resistant PC-9 cells (Fig. 8C), indicating potential for overcoming drug resistance.

To assess clinical relevance, we analyzed PAMT-001 sensitivity in 29 AML patients (Fig. 8D). LD_50_ values showed no significant variation with gender, AML risk classification, or remission status, suggesting broad efficacy (Fig. 8E). Notably, NPM1-mutated AML cells were more sensitive to PAMT-001, with a lower LD_50_ and reduced odds of resistance (Fig. 8F, G; odds ratio: 0.1572, *p* = 0.0058). GSEA revealed that NPM1-mutated AML cells had elevated mtOXPHOS gene signatures (Fig. 8H), along with an increased *ESRRA* expression (Fig. 8I). Overall, PAMT-001 inhibited tumor growth without causing toxicity in vivo, supporting the therapeutic efficacy of PAMT-001. The genetic association suggests that PAMT-001 is particularly effective in treating NPM1-mutated AML by controlling high OXHPOS status, highlighting its clinical potential.

## Discussion

We report that PAMT-001, a new ERRα antagonist, induces multiple cell death pathways, named ‘PAAoptosis’, including pyroptosis, apoptosis, and autophagic cell death, across multiple types of cancers, including drug-resistant cancers. PAMT-001 effects upon suppression of mitochondrial complexes I and III lead to mtROS generation, resulting in tumor cell death. ER stress gene CHOP is also involved in PAMT-001-mediated cell death. The CHOP signaling pathway is associated with apoptosis, ferroptosis, and both immunogenic and autophagic cell death in various cancers [59–61]. Although the precise relationship between mtROS and CHOP activation remains unclear, PAMT-001-induced mtROS appears to amplify ER stress, thereby enhancing cell death. According to the Goldie–Coldman hypothesis, the emergence of drug-resistant cancer cells is driven by random mutations, particularly in tumors with a high cellular burden, thereby accelerating disease progression and therapeutic failure [62]. To prevent drug resistance, tumor burden must be reduced by activating multiple cell death pathways. In this regard, PAMT-001 demonstrates promising therapeutic potential by simultaneously triggering multi-modal anticancer responses. PAMT-001 induces excessive, cytotoxic autophagy alongside mitochondrial dysfunction, likely due to overwhelming cellular stress that surpasses the adaptive threshold. Moreover, PAMT-001 activates caspase-3, which cleaves GSDME, leading to the formation of membrane pores. These pores disrupt plasma membrane integrity and facilitate mitochondrial swelling and permeabilization, thereby amplifying cell death signals [54–56]. These results showed that PAMT-001’s anticancer effects arise from its capacity to coordinate and amplify several interconnected forms of regulated cell death. Accordingly, PAMT-001 emerges as a strong candidate for further development as a multi-modal anticancer agent capable of effectively targeting treatment-resistant tumors.

Patient outcomes in metastatic solid tumors and hematologic malignancies are often compromised by the persistence of residual disease following treatment, a hallmark of therapy resistance. Our study demonstrated that PAMT-001, a novel mitochondrial-targeting compound, effectively induces cell death across multiple cancer types, including colon cancer, lung cancer, and chemoresistant AML. Importantly, PAMT-001 was able to overcome gefitinib resistance in lung cancer, highlighting its potential as a second-line or combination therapy in EGFR-targeted treatment failure. It also selectively eliminated AML cells while sparing normal hematopoietic mononuclear cells, suggesting a favorable therapeutic index. This selectivity is likely due to the elevated mtOXPHOS activity observed in gefitinib-resistant cancer cells [63] and AML blasts [64–66], which distinguishes them metabolically from normal cells. PAMT-001 exhibited broad efficacy across genetically diverse AML subtypes, with particularly pronounced effects in NPM1-mutated AML cells, which are characterized by elevated mtOXPHOS. These findings highlight the potential of PAMT-001 as a precision medicine approach for targeting metabolically reprogrammed cancer cells. By exploiting the mitochondrial vulnerabilities of therapy-resistant cancer cells, PAMT-001 may effectively reduce residual disease and prevent relapse. However, further research is needed to elucidate the underlying mechanisms linking NPM1 mutations to mitochondrial function and PAMT-001 sensitivity.

In summary, PAMT-001—a novel anticancer agent targeting ERRα—represents a promising therapeutic strategy that combines mitochondrial targeting, resistance reversal, and selective cytotoxicity. Its ability to induce PAAoptosis, a multi-modal cell death pathway, not only enhances its anticancer efficacy but also reduces the likelihood of therapeutic resistance and escape. Its preferential activity in metabolically active and genetically defined cancer subsets, such as NPM1-mutated AML, supports its development as a personalized treatment option.

## Materials and methods

(See Supplementary Information for more)

### Cell lines and chemicals

THP-1, HL-60, and 5637 cell lines were purchased from the Korean Cell Line Bank (Seoul, Korea), and K562-Luci, KG1α, HCT-116, HT-29, A549, PC-9, and MC38 were kindly provided by Prof Heo, Prof Song, and Prof Jung (CNU, Korea). PAMT-001 is provided by Prof. Ahn’s laboratory (Gwangju Institute of Science and Technology). The detailed information is provided in the Supplementary Materials.

### Docking model between PAMT-001, PGC1α, and ERRα

3D structures of ERRα and PGC1α (PDB: 1XB7) and prediction of their binding affinity were drawn using PyMOL (version 3.1.4; Schrödinger, LLC) and Molmoda. The detailed method is provided in the Supplementary Materials.

### ERRα coactivator TR-FRET assay

ERRα coactivator TR-FRET assay was performed using the ERRα ligand-binding domain (LBD) and a fluorescein-labeled coactivator peptide. The detailed method is provided in the Supplementary Materials.

### Patient samples and cell preparation

After informed consent, Chungnam National University Hospital’s IRB (CNUH2018-08-013-012) took bone marrow or peripheral blood samples for diagnostic purposes. All experiments were carried out following the Helsinki Declaration. A detailed explanation of the preparation of primary AML cells is provided in the Supplementary Materials.

### RNA sequencing

Total RNA was extracted using Trizol reagent (Invitrogen) and analyzed by NovaSeq 6000 (Illumina, Inc., USA). The detailed procedures are provided in the Supplementary Materials.

### Animal experiments with tumor xenograft

GHbio (Daejeon, Korea) provided NOD/SCID/IL2Rnull (NIG) mice. Koatech (Pyeongtaek, Korea) provided the athymic and NOD/SCID mouse. The Institutional Animal Care and Use Committee reviewed and authorized all in vivo research, and all animals (6~8 weeks) were maintained in a pathogen-free environment (CNUH-020-A0054, CNUH-2022-A0010-00, CNU IACUC-H-2024-48). Detailed descriptions of animal experimentation methods and in vivo bioluminescence Imaging on the IVIS platform are provided in the Supplementary Materials.

### Transfection of small interfering RNA targeting CHOP

Transfections were performed in HL-60 and 293T cells using Lipofectamine 3000 with small interfering RNA oligonucleotides targeting *CHOP* or negative control *RNA* oligonucleotides, purchased from BIONEER (AccuTarget™ Predesigned si*RNA*, Korea), according to the manufacturer’s protocols. HL-60 cells were centrifuged at 200 × *g* for 60 min with polybrene (10 µg/mL) for spinoculation at room temperature after loading Lipofectamine 3000 with si*RNA*.

### RNA extraction and real-time quantitative PCR (qRT-PCR)

RNA extraction and real-time quantitative PCR were performed as described previously [67, 68], and detailed explanations are provided in the Supplementary Materials.

### Western blot

Cells were lysed in RIPA buffer and were denatured in SDS sample buffer, separated by SDS–PAGE, transferred to PVDF membranes, blocked in TBST, incubated with primary and secondary antibodies, and visualized by ECL chemiluminescence. The western blot antibodies are listed in the Supplementary Data.

### Flow cytometry for apoptosis quantitation, MitoSOX, and dextran-FITC uptake analysis

The quantitation of apoptosis, MitoSOX, and dextran-FITC uptake analysis was confirmed by flow cytometry (FACSCanto II or NovoCyte flow cytometer) using FITC Annexin V apoptosis detect Kit (BD Bioscience, Cat# 556547) or annexin V-APC (BD Bioscience, Cat# 561012), propidium iodide (PI) (BD Bioscience, Cat# 556463), MitoSOX (Invitrogen, Cat# M36008), and dextran-FITC (Sigma, Cat# FD4-100MG). Detailed explanations are provided in the Supplementary Materials.

### Statistical analysis for in vitro and in vivo data

The statistical analysis for in vitro and in vivo data was done with the SPSS or Prism software (GraphPad 10.2.0). According to the normality test, a two-tailed *t*-test or a non-parametric test was used to compare the two conditions. *P* < 0.05 (*), *P* < 0.01 (**), *P* < 0.001 (***), and *P* < 0.0001 (****) were used to determine statistically significant differences.

Other methods are described in detail in the supplementary method.

## Supplementary information

Supplementary material & information

Full and uncropped western blots

## Data Availability

All data are available upon request or in supplementary files.
