# Supplementary material for: A novel PAAoptosis-inducing ERRα-targeting compound for combating hematopoietic and solid cancers
Source: Cell Death Discov. 2026 Mar 26;12:188. doi: 10.1038/s41420-026-03010-4 (PMC13139410; doi:10.1038/s41420-026-03010-4)
Supplement: Supplementary file 1 — Supplementary material & information [file 41420_2026_3010_MOESM1_ESM.docx]

**Supplementary Information for**

**A novel PAAoptosis-inducing ERRα-targeting compound for combating hematopoietic and solid cancers**

Wonhyoung Seo^1,2#^, Yerim Heo^3#^, Khang Vuong Tran^4#^, So-young Kim^4^, Eun Jung Bae^5^, Bokeum Jung^6^, Sang-Hee Lee^7,8^, Taylor Roh^1^, Sang Min Jeon^1^, Kyung Tae Kim^1^, Eun-Jin Park^1^, Soo In Kim^1^, Jeong Suk Koh^2^, Ik-Chan Song^2^, Hyun Kyu Song^9^, Jung-Joon Min^4*^, Jin Hee Ahn^3*^, and Eun-Kyeong Jo^1*^

^#^These authors contributed equally to this work. *Corresponding authors e-mail: [jjminmd@gmail.com](mailto:jjminmd@gmail.com); [jhahn@gist.ac.kr](mailto:jhahn@gist.ac.kr); [hayoungj@cnu.ac.kr](mailto:hayoungj@cnu.ac.kr).

**This file includes:**

Supplementary Methods

Supplementary Figure and Figure Legends

Supplementary Table

1. **Supplementary Methods**

**Synthesis of PAMT-001**

All solvents and chemicals were used as purchased without further purification. All reported yields are isolated yields after column chromatography or crystallization. ^1^H NMR spectra and ^13^C spectra were recorded on a JEOL JNM-ECS400 spectrometer at 400 MHz for ^1^H NMR and 100 MHz for ^13^C NMR, respectively. The chemical shift (δ) is reported in ppm relative to tetramethylsilane (TMS) as an internal standard, and chloroform-*d* and DMSO-*d_6_* were used as solvents. Multiplicity of peaks is reported as s (singlet), d (doublet), t (triplet), q (quartet), dd (doublet of doublets), td (triplet of doublets), qd (quartet of doublets), dt (doublet of triplets), dq (doublet of quartets), ddd (doublet of doublets of doublets), and m (multiplet). High-performance liquid chromatography (HPLC) analyses were performed with a Waters Agilent HPLC system equipped with a PDA detector and a Waters SB-C18 column (1.8 μm, 2.1 × 50 mm^2^). The mobile phase was used with buffer A (ultrapure H_2_O containing 0.1% trifluoracetic acid) and buffer B (chromatographic-grade CH_3_CN) for method. The flow rate was 0.5 mL/min.

The synthesis of (E)-2-(4-((2,4-bis(trifluoromethyl)benzyl)oxy)-3-methoxybenzylidene)hydrazine-1-carboximidamide hydrochloride (PAMT-001) was carried out according to the following procedure.

*Step 1.* A mixture of 2,4-Bis(trifluoromethyl)benzyl bromide 1 (2.0 g, 6.51 mmol), vanilin (1.19 g, 7.82 mmol), and potassium carbonate (2.25 g, 16.29 mmol) in acetone (20 mL) was stirred under reflux for 18 h. After the reaction mixture was cooled to room temperature, the volume was reduced by evaporation and water was added to the resulting mixture, followed by extraction with ethyl acetate. The combined organic phase was dried over anhydrous sodium sulfate, filtered, and evaporated in vacuo. The crude product was purified by silica gel column chromatography to give 4-((2,4-bis(trifluoromethyl)benzyl)oxy)-3-methoxybenzaldehyde 3 (2.18 g, 89 %) as a white solid. ^1^H NMR (400 MHz, chloroform-*d*) δ 9.87 (s, 1H), 7.99-7.96 (m, 2H), 7.86 (d, *J* = 8.2 Hz, 1H), 7.48 (d, *J* = 1.8 Hz, 1H), 7.42 (dd, *J* = 8.2, 1.8 Hz, 1H), 6.94 (d, *J* = 8.2 Hz, 1H), 5.47 (s, 2H), 3.99 (s, 3H). LC-MS (m/z): 311.5 [M+H]^+^ .

*Step 2.* To a solution of 4-((2,4-bis(trifluoromethyl)benzyl)oxy)-3-methoxybenzaldehyde 3 (50 mg, 0.13 mmol) in methanol (5 mL) were added aminoguanidine hydrochloride (16 mg, 0.15 mmol), and the resulting mixture was heated to reflux for 18 h. After cooling to room temperature, the mixture was concentrated and the residue was collected by filtration, and washed with methanol to afford (E)-2-(4-((2,4-bis(trifluoromethyl)benzyl)oxy)-3-methoxybenzylidene)hydrazine-1-carboximidamide hydrochloride (PAMT-001) (59 mg, 95 %) as a white solid. ^1^H NMR (400 MHz, DMSO-*d_6_*) δ 11.79 (s, 1H), 8.17 (d, *J* = 8.7 Hz, 1H), 8.10 (s, 1H), 8.09 (s, 1H), 8.03 (d, *J* = 8.2 Hz, 1H), 7.62 (d, *J* = 1.9 Hz, 1H), 7.27 (dd, *J* = 8.3, 2.0 Hz, 1H), 7.08 (d, *J* = 8.3 Hz, 1H), 5.38 (s, 2H), 4.68 (s, 1H), 3.86 (s, 3H); ^13^C NMR (100 MHz, DMSO-*d_6_*) δ 155.41, 149.54, 149.21, 146.50, 139.89, 131.05, 129.93, 129.91, 129.85, 127.40, 123.09, 123.06, 123.03, 122.26, 113.32, 109.57, 66.12, 55.94; HPLC purity 94.75 %.

**Cell culture**

Cells were cultured in RPMI 1640 medium (THP-1, HL-60, 5637, K562-Luci, KG1α, and PC9) (Lonza, Cat# 12-702F) or DMEM (HCT116, HT-29, A549, and MC38) (Lonza, Cat# 12-604F) supplemented with 10% (20% for HL-60) fetal bovine serum (FBS)(Gibco, Cat# 16000-044) and 50U/ml penicillin/streptomycin(Lonza, Cat# 17-745E) at 37 degree in a humidified 5% CO_2_ incubator. The cultured cells were maintained in an exponential growth phase by splitting every 2-3 days.

**Patient samples and cell preparation**

Primary AML cells were obtained from bone marrow or peripheral blood at the time of diagnosis. Ficoll-Paque gradient ultracentrifugation (Stemcell, Cat# 07851) was used to isolate human AML blast cells and mononuclear cells; isolated mononuclear cells were then treated with RBC lysis buffer (Invitrogen, Cat# 501129757) to remove red blood cells. The cells were stored in liquid nitrogen after being frozen in Cell freezing media (Nacalai tesque, Kyoto, Japan, Cat# 07485-44). Flow cytometry and morphologic characteristics were used to determine the percentage of blast cells in the patient's samples before blast cell purification. All patients' blast cells were cultured in 10% FBS with 5% supernatant from the 5637-bladder cancer cell line (to supply interleukin-1 beta, granulocyte-macrophage colony-stimulating factor, granulocyte colony-stimulating factor, macrophage colony-stimulating factor, and stem cell factor) [1-3].

**Prediction assay of the binding affinity between ERRα and PGC1α**

3D structures of ERRα and PGC1α (PDB: 1XB7) and prediction of their binding affinity were drawn using PyMOL (version 3.1.4; Schrödinger, LLC)**.** Electrostatics of the protein were assessed with the Adaptive Poisson-Boltzmann Solver (APBS) [4]. Polar contacts between the ligand and protein were calculated using PyMOL. Before analysis, the protein complex was prepared by adding hydrogens and optimized after protonation [5,6]. Binding pockets were assessed through Fpocketweb and DoGSiteScorer, filtered with the drugabillity score [7,8]. AutoDock Vina built-in Molmoda was used to simulate and evaluate GM91218 binding with ERRα [9-11].

**ERRα coactivator TR-FRET assay**

ERRα coactivator TR-FRET assay was performed using the ERRα ligand binding domain (LBD) and a fluorescein-labeled coactivator peptide. Compounds were serially diluted in DMSO (eight concentrations) and incubated with 2.5 nM ERRα-LBD in TR-FRET Coregulator buffer G (1% DMSO final). After 60 minutes of incubation at 25 °C, the amount of ERRα-LBD/coactivator complex formed was determined by measuring the TR-FRET signal using a spectrofluorometer at 520 nm/490 nm. A ≥50% fluorescence reduction compared to 3 μM XCT790 (positive control) indicated inverse agonism. EC50 values were calculated by fitting the data to sigmoidal dose-response curves.

**Cell viability & LD_50_ measurements**
Cells were seeded on 96-well plates at 70% confluency, and viability assays were performed by adding 10% CCK-8 (DOJINDO, Cat# CK04) or MTT solution for 2~4 hours at each condition. The optical density at 450nm for CCK-8 assay or at 590nm for MMT assay was measured using a microplate spectrophotometer. Absolute O.D value or cell count from each condition was converted to percentage viability versus the vehicle control. Each condition's absolute O.D value or cell count was translated to percentage viability versus the vehicle control. Based on the cell viability assay, a non-linear fit of 'inhibitor versus response (three parameters)' was performed in GraphPad Prism v8.0 to generate Lethal dosage 50 (LD_50_) values.

The ratio of live and dead cells was determined on the indicated days by counting the cells in a hemocytometer using the trypan blue exclusion method.

**Transfection of small interfering RNA targeting CHOP**

Transfections were performed in HL-60 and 293T cells using Lipofectamine 3000 with small interfering RNA oligonucleotides targeting on *CHOP* or negative control *RNA* oligonucleotides, purchased from BIONEER (AccuTarget™ Predesigned si*RNA*, Korea), according with the manufacturer's protocols. HL-60 cells were centrifuged at 200x g for 60 minutes with polybrene (10 µg/mL) for spinoculation at room temperature after loading Lipofectamine 3000 with si*RNA*,.

**Flow cytometry for apoptosis quantitation**

The quantitation of apoptosis was confirmed by flow cytometry (FACSCanto II or NovoCyte flow cytometer) using FITC Annexin V apoptosis detect Kit (BD Bioscience, Cat# 556547) or annexin V-APC (BD Bioscience, Cat# 561012) and propidium iodide (PI) (BD Bioscience, Cat# 556463) following manufacturer’s protocol.

**Flow cytometry for MitoSOX and dextran-FITC uptake analysis**

The quantitation of mitochondrial reactive oxygen species and dextran uptake examination was confirmed by flow cytometry (FACSCanto II). Using MitoSOX (Invitrogen, Cat# M36008) were used to detect mitochondrial reactive oxygen species following STAR protocol [12]. For analysis of pyroptosis, dextran-FITC (Sigma, Cat# FD4-100MG) was diluted with RPMI-1640 (final concentration: 0.25g/ml) and incubated in cells for 6 hours. After then, the cells were then washed twice with PBS and assessed using flow cytometry to detect intracellular dextran.

**RNA extraction and real-time quantitative PCR (qRT-PCR)**

RNA extraction and real-time quantitative PCR were performed using TRizol reagent (Invitrogen), Reverse transcriptase kit (Elpis, Daejeon, South Korea), and SYBR Green PCR Kits (Qiagen) in the Real-time PCR cycler Rotor-Gene Q 2plex system (Qiagen). Data were analyzed using the 2^-ΔΔCt^ method to assess relative expression of target RNA with human GAPDH as a control gene, and RNA expression is displayed as relative fold changes. The information on the primers used for qPCR is summarized in the supplementary data.

**RNA sequencing**

After extracting total RNA using Trizol reagent (Invitrogen), the quality of extracted RNA was assessed using an Agilent 2100 bioanalyzer (Agilent Technologies, Amstelveen, Netherlands), and RNA quantification was performed using an ND-2000 Spectrophotometer (Thermo Inc., DE, USA). Libraries were generated from total RNA using the NEBNext Ultra II Directional RNA-Seq Kit (NEW ENGLAND BioLabs, Inc., UK). The mRNA was obtained using the Poly(A) RNA Selection Kit (LEXOGEN, Inc., Austria) and subsequently converted into cDNA and subjected to shearing. Indexing was conducted utilizing the Illumina indexes. High-throughput sequencing was conducted as paired-end 100 sequencing using the NovaSeq 6000 (Illumina, Inc., USA). Read counts for each RefGene symbol from the UCSC database were calculated using the htseq-count function in SAMtools16. DEseq217 was used to identify differentially expressed genes between control and PAMT-001-treated cells (FDR 1.5)

**Animal experiments with tumor xenograft**

HCT116 cells were subcutaneously injected in athymic mouse or Balb/c nu-/nu mouse respectively for measuring gross tumor mass (5 x 10^6^ cells in 100 µL of Hank’s Balanced Salt Solution). After tumor mass was found, tumor volumes were estimated every 2~3 days using the tumor length (a) and width (b) measured with a digital caliper and the formula $V=1/2\left( a\times b^{2} \right)$.

HL-60 cells were intravenously injected (4 x 10^6^ cells in 100 µL of Hank’s Balanced Salt Solution) into the tail vein of NOD/SCID mice 24 hours after radiation (250cGy) to assess the therapeutic potential of PAMT-001 in orthotopic models. On the seventh day following xenotransplantation, PAMT-001 (6mg/kg) was administered intraperitoneally every 2 days. After 3 weeks, mice were killed, and engrafted HL-60 cells in mouse bone marrow were obtained and examined using flow cytometry with anti-human CD45 (BD Biosciences, Cat# 555485) and anti-mouse CD45 antibodies (BD Biosciences, Cat# 553081).

For IVIS animal models, we used K562 Red F-luc Bioluminescent Tumor Cell line (K562-luc) which was kindly given by Prof. Song, (CNU, Korea). K562-luc cells were intravenously injected (4 x 10^6^ cells in 100 µL of Hank’s Balanced Salt Solution) through the tail vein in NOD/SCID/Il2rg null (Koatech). PAMT-001 (6mg/kg) was treated via the intraperitoneal route once every 2 days on the 7th day after confirming engraftment of K562-luc cells. Tumor burden was assessed using the IVIS system once a week until 4 weeks after K562-luc cell transplantation.

**Mitochondrial Respiration**

The oxygen consumption rate (OCR, pmol/min/unit) was calculated using the XFe96 (THP-1: 5 x 10^5^ cells/mL, KG1α: 1 x 10^5^ cells/mL) analyzers according to the manufacturer's protocol. Beginning with baseline conditions and progressing through serial injections of Oligomycin (an ATPase inhibitor, final concentration 2µg/ml), CCCP (an uncoupler, final concentration 5µM), and Rotenone/Antimycin (mitochondrial complex I inhibitor, final concentration 2µM), oxygen consumption was monitored over time. The tests were run on the same plate at the same time.

**Immunofluorescence analysis**

Cells were grown on cover slips in 24-well plates followed by PAMT-001 treatment (3.75μM) for 12h. After treatment, the cells were washed twice with PBS followed by fixation in 4% paraformaldehyde for 10min. Then, the cells were treated with 0.25% triton X-100 for permeabilization and incubated overnight at 4 degrees with MitoSOX (2ug/mL) or GSDME antibody (1:250 diluted) (Abcam, Cat# ab214191). The cells were washed three times with DPBS before being incubated with the secondary antibody for two hours at room temperature. To stain and mount the cells, Fluoromount-G with DAPI (Waltham, Cat# 00-4958-02) was used. Prepared samples were detected by confocal microscopy.

**In vivo bioluminescence Imaging on IVIS platform**

Intraperitoneal injection of 150 mg/kg body weight of IVISbrite D-luciferin potassium salt bioluminescent substrate (PerkinElmer, Cat# 122799), the substrate of luciferase, was injected into mice. The mice were positioned on the imaging stage of the IVIS apparatus in the abdominal position after being anesthetized with 1.5% isofluranein and 100% O2. Using the IVIS Imaging system (Lumina XRMS instrument (PerkinElmer)), images were taken within 10 minutes after the luciferin injection. Photons emitted from the tumor and its surroundings were measured through the Aura imaging software (Spectral Instruments Imaging, USA).

**Electron microscopy and morphology**

The cells were rinsed in PBS before being fixed for 3 hours in 0.1 M sodium cacodylate buffer (pH 7.2) containing 0.1% CaCl2. The samples were then post-fixed for 2 hours in 0.1 M sodium cacodylate buffer with 0.1% CaCl2, rinsed with cold distilled water, and gently dehydrated at 4°C with a series of ethanol concentrations and propylene oxide. The samples were embedded with Embed-812 (EMS; 14120) and then cured at 60°C for 36 hours. The ultrathin sections (70-80 nm) were cut using a ULTRACUT UC7 ultramicrotome (Leica, Germany) with a diamond knife placed on formvar-coated slot grids. Sections were stained for 10 minutes with 4% uranyl acetate and 7 minutes with lead citrate. To scan stained sections, a KBSI Bio-High Voltage EM system (JEM-1400 Plus and JEM-1000 BEF; JEOL Ltd., Tokyo, Japan) was used. Cristae width was assessed for the morphometric study of mitochondrial cristae using Image J's Multimeasure plug-in [13].

**Information of reagents and antibodies used in this study**

| **Reagents** | **Source** | **Cat# and Identifier** |
| --- | --- | --- |
| **Antibodies** | | |
| Rabbit polyclonal anti-caspase-3 antibody (1:1,000) | Cell Signaling Technology | Cat#9662;  RRID: AB_331439 |
| Rabbit polyclonal anti-cleaved Caspase-3 (Asp175) antibody (1:1,000) | Cell Signaling Technology | Cat#9661 ;  RRID: AB_2341188 |
| Mouse monoclonal anti-caspase-9 (C9) antibody (1:1,000) | Cell Signaling Technology | Cat# 9508;  RRID:AB_2068620: |
| Rabbit Cleaved Caspase-9 (Asp315) Antibody (1:1,000) | Cell Signaling Technology | Cat# 9505 |
| Mouse monoclonal anti-PARP antibody (1:1000) | BD Biosciences | Cat# 556362;  RRID:AB_396387 |
| Mouse monoclonal ATP5A1 Antibody (7H10BD4F9) (1:1,000) | Thermo Fisher Scientific | Cat# 459240;  RRID:AB_2532234 |
| Rabbit polyclonal COX IV Antibody (1:1,000) | Cell Signaling Technology | Cat# 4844;  RRID: AB_2085427 |
| Rabbit monoclonal anti-ERRa (E1G1J) antibody (1:1,000) | Cell Signaling Technology | Cat# 13826; |
| Mouse total OXPHOS blue native WB antibody (1:1,000) | Abcam | Cat# ab110412;  RRID:AB_2847807 |
| Rabbit monoclonal anti- β-Actin (13E5) (HRP Conjugate) antibody (1:1,000) | Cell Signaling Technology | Cat# 5125; |
| Goat polyclonal anti-Actin (I-19) antibody (1:5,000) | Santa Cruz Biotechnology | Cat# sc-1616;  RRID:AB_630836: |
| Donkey anti-goat IgG-HRP antibody (1:5,000) | Santa Cruz Biotechnology | Cat# sc-2020;  RRID:AB_631728_ |
| Horse anti-mouse IgG-HRP antibody (1:5,000) | Cell Signaling Technology | Cat# 7076;  RRID:AB_330924 |
| Goat anti-rabbit IgG-HRP antibody (1:5,000) | Cell Signaling Technology | Cat# 7074  RRID:AB_2099233 |
| PE Rat Anti-mouse CD45 | BD Biosciences | Cat# 553081;  RRID: AB_394611 |
| APC Mouse Anti-Human CD45 | BD Biosciences | Cat# 555485  RRID:AB_398600 |
| **Chemicals** | | |
| Propidium Iodide | BD biosciences | Cat# 556463 |
| Annexin V FITC Apoptosis Detection Kit I | BD biosciences | Cat# 556547 |
| APC Annexin V | BD biosciences | Cat# 561012 |
| N-Acetyl-L-cysteine (NAC) | Sigma Aldrich | Cat# A9165 |
| Z-VAD-FMK | MedChemExpress | Cat# HY-16658B |
| Q-VD-OPh | MedChemExpress | Cat# HY-12305 |
| Mito Q | Sigma Aldrich | Cat# SML3152 |
| Polybrene | Santa Cruz Biotechnology | Cat# NC9840454 |
| Dextran-FITC | Sigma Aldrich | Cat# FD4 |
| IVISbrite D-luciferin potassium salt bioluminescent substrate | PerkinElmer | Cat# 122799 |
| XCT-970 | Sigma Aldrich | Cat# X4753 |
| Lipofectamine™ 3000 Transfection Reagent | Thermo Fisher Scientific | Cat# L3000-008 |
| **Assays, primers and probes for qPCR** | | |
| *COX5A:*  Fwd 5’-GATGCTCGCTGGGTAACATA-3’  Rev 5’-GGGCTCTGGAACCATATCAT-3’ |  | [NM_004255.4](https://www.ncbi.nlm.nih.gov/entrez/viewer.fcgi?db=nucleotide&id=1519244926) |
| *COX5B*  Fwd 5’-AAAGAAGGGACTGGACCCATA-3’  Rev 5’-CAGACGACGCTGGTATTGTC-3’ |  | [NM_001862.3](https://www.ncbi.nlm.nih.gov/entrez/viewer.fcgi?db=nucleotide&id=1653962030) |
| *NDUFS3*  Fwd 5’-CCTGTTGTCTCTGCGCTTCAA-3’  Rev 5’-GAAGACTCCAAACATGTCCCAG-3’ |  | [NM_004551.3](https://www.ncbi.nlm.nih.gov/entrez/viewer.fcgi?db=nucleotide&id=1519315626) |
| *UQCRFS1*  Fwd 5’-CCTGTGTTGGACCTGAAGC-3’  Rev 5’-ATAACAAACAGAAGCAGGGACAT-3’ |  | [NM_006003.3](https://www.ncbi.nlm.nih.gov/entrez/viewer.fcgi?db=nucleotide&id=1519315257) |
| *CHOP*  Fwd 5’-CAAGATCATCAGCAATGCCTCC-3’  Rev 5’-GGTCATGAGTCCTTCCACGA-3’ |  | NM_004083.6 |
| *EDEM*  Fwd 5’-CAAGTGTGGGTACGCCACG-3’  Rev 5’-AAAGAAGCT CTCCATCCGGTC-3’ |  | NM_014674.3 |
| *HSPA5*  Fwd 5’-TGTTCA ACCAATTATCAGCAA ACTC-3’  Rev 5’-TTCTGCTGTATCCTCTTC ACC AGT-3’ |  | NM_005347.5 |
| *ATF4*  Fwd 5’-GTTCTCCAGCGACAAGGCTA-3’  Rev 5’-ATCCTGCTTGCTGTTGTTGG-3’ |  | NM_001675.4 |
| *GAPDH*  Fwd 5’-CAAGATCATCAGCAATGCCTCC-3’  Rev 5’-GGTCATGAGTCCTTCCACGA-3’ |  | [NM_001357943.2](https://www.ncbi.nlm.nih.gov/entrez/viewer.fcgi?db=nucleotide&id=1676440496) |
| Software | | |
| FlowJo v10.7.1 | BD biosciences | RRID:SCR_008520 |
| Prism 8 | GraphPad | RRID:SCR_002798 |
| Adobe illustrator 2021 | Adobe | RRID:SCR_010279 |
| IBM SPSS statistics 26 | IBM | RRID:SCR_016479 |
| ImageJ 1.43u | https://imagej.net/ | RRID:SCR_003070 |
| R v. 4.1.1 | The R foundation | RRID:SCR_001905 |
| Others | | |
| Seahorse XF Base Medium | Seahorse Bioscience | Cat# 103334 |
| Oligomycin A | Sigma Aldrich | Cat# O4876 |
| Carbonyl cyanide 3-chlorophenylhydrazone | Sigma Aldrich | Cat# C2759 |
| Rotenone | Sigma Aldrich | Cat# R8875 |
| RPMI-1640 | Lonza | Cat# 12-702F |
| DMDM | Lonza | Cat# 12-604F |
| Pen/Strep amphotericin B | Lonza | Cat# 17-745E |
| Fetal bovine serum | Gibco | Cat# 16000-044 |
| Cell Reservoir One (with DMSO) | Nacalai tesque | Cat# 07485-44 |
| HBSS | Welgene | Cat# LB003 |
| Reverse Transcriptase Premix (oligo d(T) _15_) | ELPis | Cat# EBT-1515C |
| Quantinova® SYBR® Green PCR kit (2500) | Qiagen | Cat# 208056 |
| Lymphoprep | Stemcell | Cat# 07851 |
| 1X RBC lysis buffer | Invitrogen | Cat# 501129757 |

1. **Supplementary Figure and Figure Legends**

**
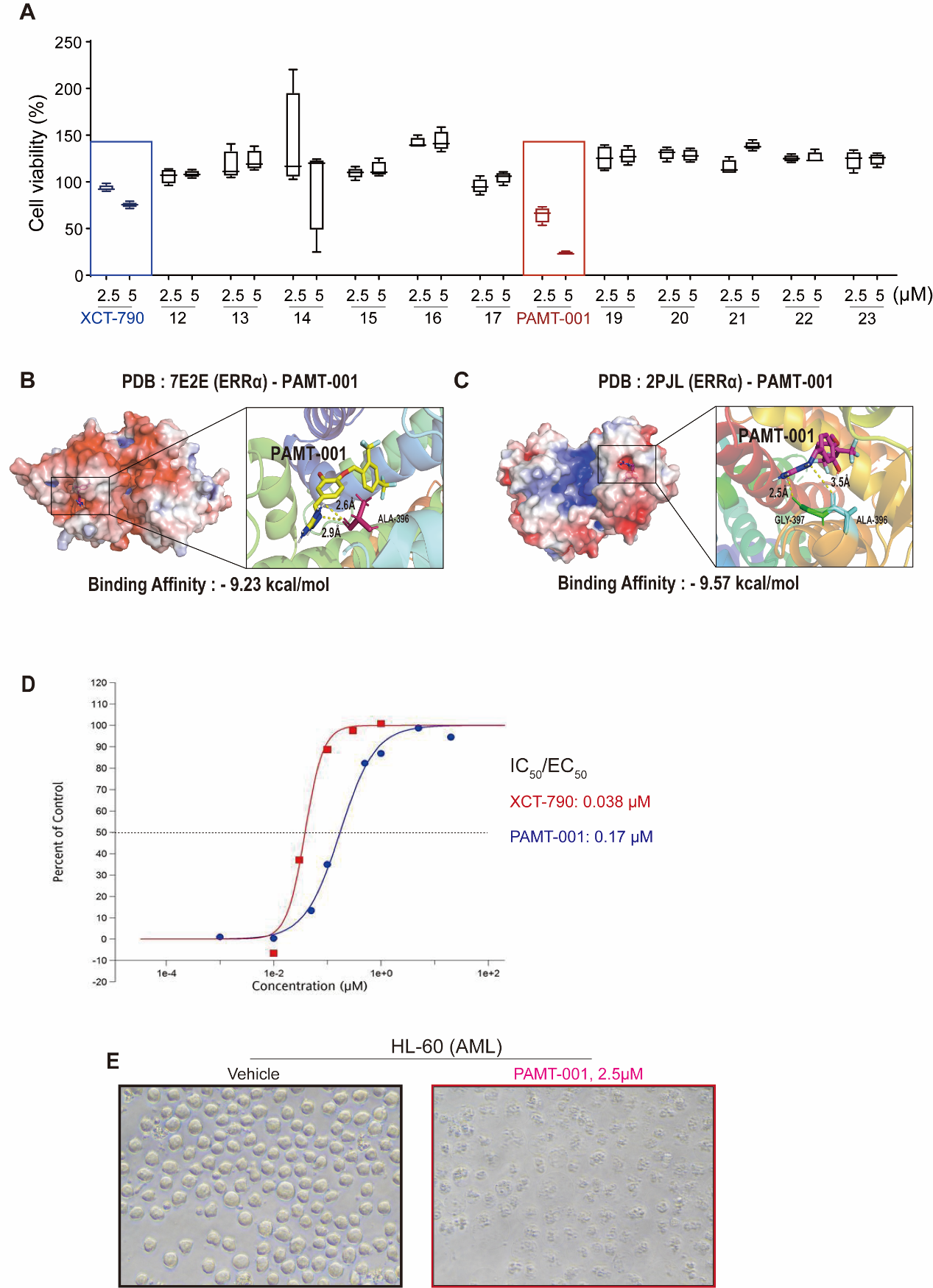
**

**Supplementary Fig. 1 PAMT-001 binds to ERRα and modulates its activity, leading to cancer cells death.**

**A** Cell viability assay of KG1α cells treated with XCT-790 and 12 compound candidates (72 h). Schematic of the two compound was compared with circles highlighting difference. **B-C** 3D structures of PAMT-001 bound to ERRα at two promising druggable binding (PDB) sites (PDB: 7E2E, (**B**)) or (PDB: 2PJL, (**C**)) using PyMOL. **D** TR-FRET ERRα coactivator assay for detecting ≥50% decrease in fluorescence relative to XCT790 response. **E** Morphological characteristics of PAMT-001-induced cell death of HL-60.

**
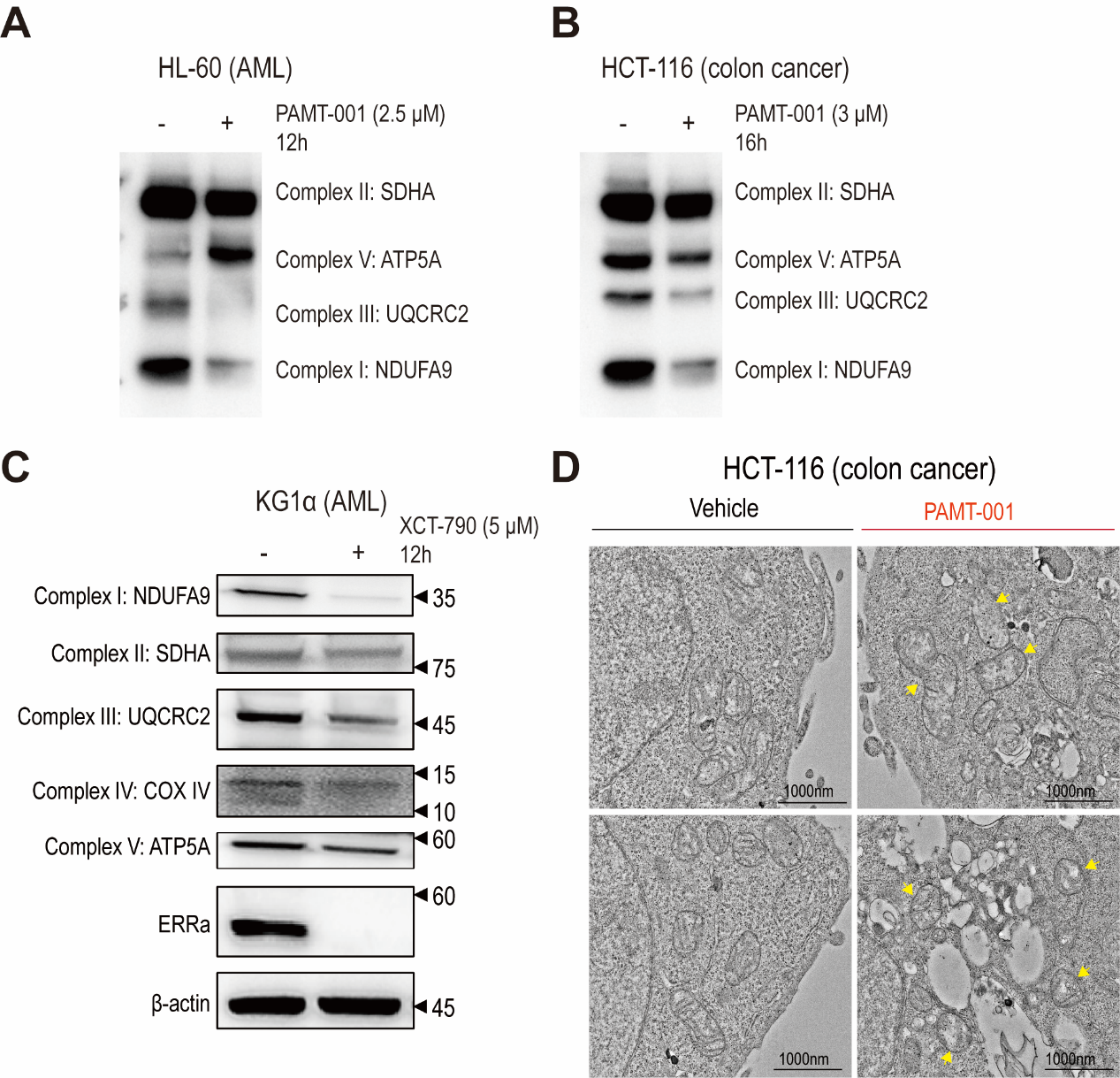
**

**Supplementary Fig. 2 PAMT-001 inhibits the protein expression related with mitochondrial oxidative phosphorylation, especially complex I and III, and districts their complexes.**

**A, B** Protein expression of OXPHOS complexes with PAMT-001 treatment in HL-60 (**A**) and HCT-116 (**B**). HL-60 and HCT-116 were treated with PAMT-001 for 12 and 16 hours, respectively. **C** Protein expression of OXPHOS complexes with XCT-790 treatment in KG1α. **D** Representative TEM showing mitochondrial distortion induced by GM91218 (2.5μM, 12h) in HCT-116.

**
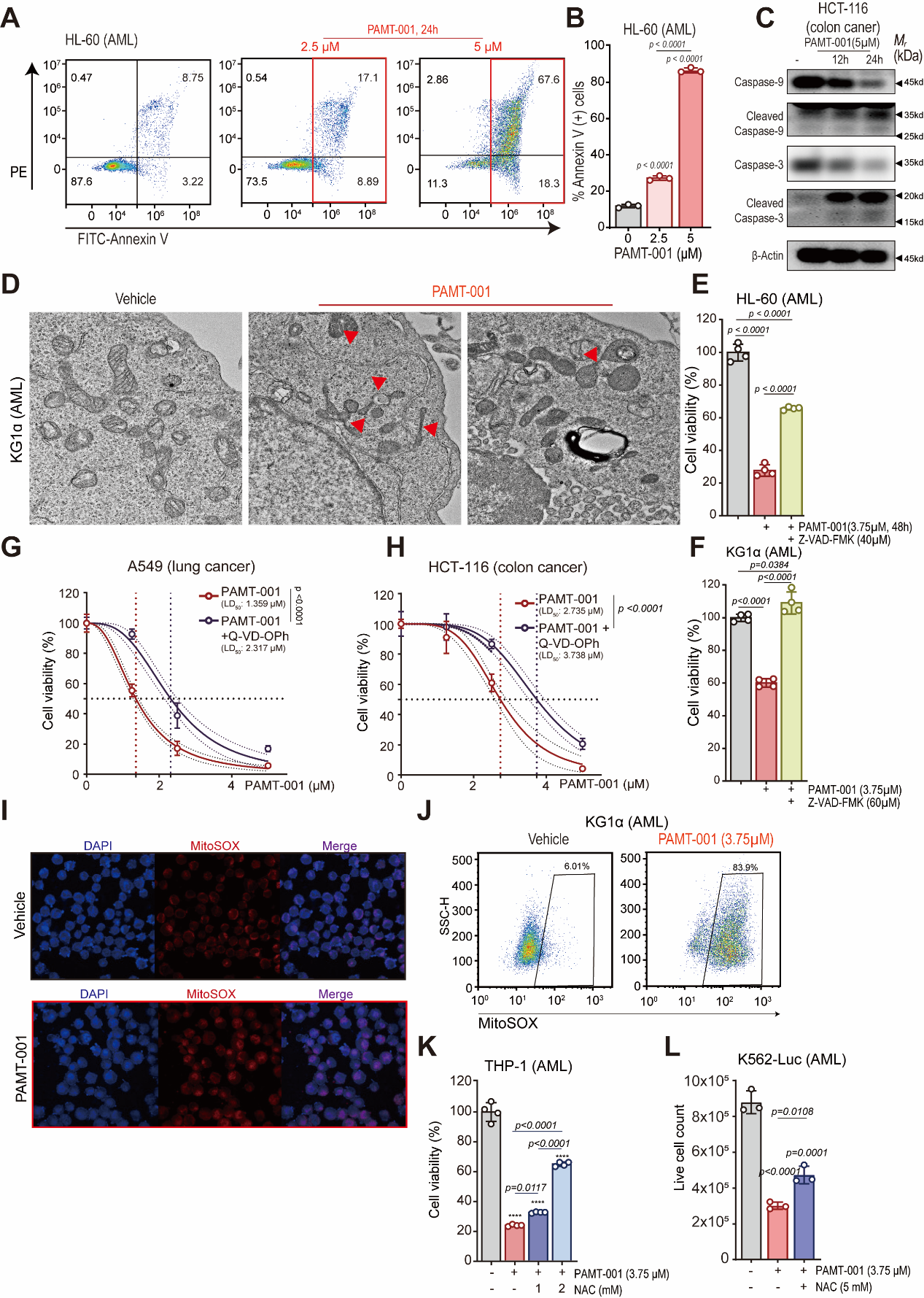
**

**Supplementary Fig. 3 PAMT-001 induces mitochondria-mediated ROS production and apoptosis.**

**A, B** Cell death measured by flow cytometry with Annexin V/PI stain in HL-60 with PAMT-001 treatment at indicated dosage (2.5 µM, 5 µM, 24 h) (**A**). The bar plot shows the fractions (%) of Annexin V positive cells (**B**). *P*-values were calculated by one-way ANOVA for multiple comparisons. **C** Western blots showing apoptosis induced by GM-9128 treatment in HCT-116. **D** Representative TEM image about mitochondria-mediated apoptosis characterized by mitochondrial fission and fragmentation (red arrowhead) in PAMT-001-treated KG1α cell. **E, F** The inhibition of the cell death induced by PAMT-001 treatment (3.75 µM) with pan-caspase inhibitor, Z-VAD-FMK (40 µM in HL-60, 60 µM in KG1α) for 24 h in KG1α or 48 h in HL-60. *P*-values were calculated by one-way ANOVA for multiple comparisons. **G, H** The LD_50_ value of PAMT-001 with and without Q-VD-OPh in lung cancer (A549) (**G**) and colon cancer (HCT-116) (**H**). The LD_50_ values were calculated through [inhibitor] vs. normalized response—Variable slope in Prism 8. *P*-value was determined by nonlinear regression. **I** Representative images of MitoSOX-stained KG1α cells (Red) were obtained by confocal microscopy. **J** Flow cytometry measuring mitochondria-derived reactive oxygen species by PAMT-001 treatment in KG1α (3.75 μM, 16 h). **K, L** PAMT-001-induced cell death measured using CCK-8 (THP-1) or trypan blue exclusion assay (K562-Luc). N-acetylcysteine (NAC) and PAMT-001 (3.75 μM) were co-treated for 48 h. *P*-values were calculated by one-way ANOVA for multiple comparisons.

**
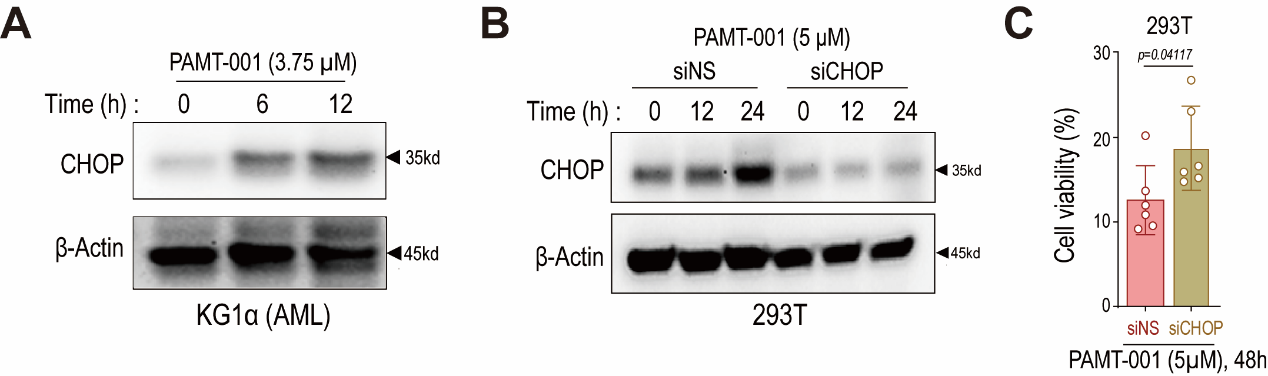
**

**Supplementary Fig. 4 Cell death caused by PAMT-001 is exacerbated by the activation of ER-stress-related genes, particularly CHOP.**

**A** Western blot analysis for PAMT-001-induced CHOP expression in KG1α. **B** The CHOP expression induced by PAMT-001 in 293T with transfection of siRNA targeting CHOP (si*CHOP*) and non-targeting (si*NS*). **C** The reduction of cell viability by PAMT-001 according to CHOP expression in 293T cells. *P-values* were calculated by two-sided *t-test* (n = 3, respectively).

**
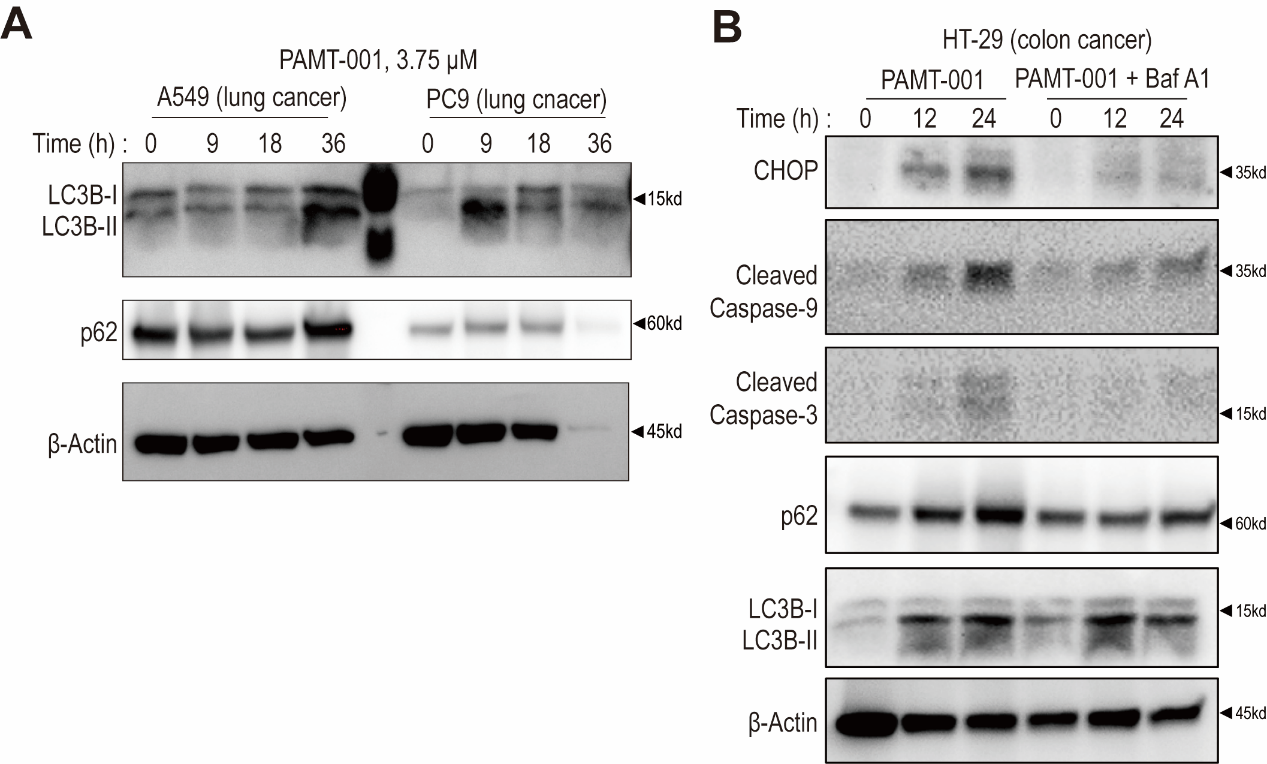
**

**Supplementary Fig. 5 Autophagy contributes PAMT-001-indcued cell death.**

**A** Western blot analysis for PAMT-001-induced autophagic flux in lung cancer cells lines (A549 and PC9). **B** Western blot analysis for autophagy-dependent cell death in HT-29. Apoptotic marker (cleaved caspase-3 and caspase-9) and CHOP expression are attenuated by bafilomycin A1 (Baf A1) treatment.

**
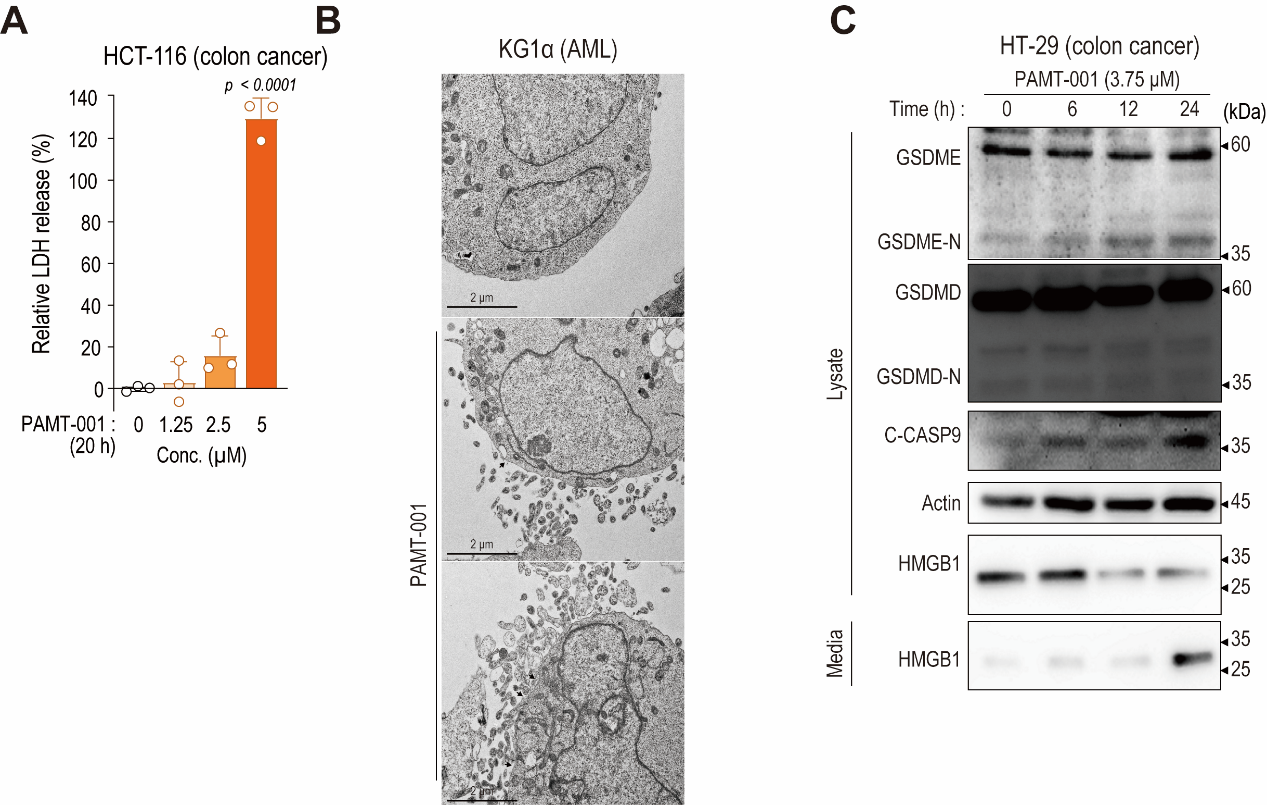
**

**Supplementary Fig. 6 PAMT-001 causes pyroptotic cell death.**

**A** Effect of PAMT-001 on LDH release from treated cells (HCT-116). *P-values* were calculated by one-way ANOVA for multiple comparisons. **B** Representative TEM images of KG1α cells treated with vehicle or PAMT-001. Disruption of the plasma membrane, which is a hallmark feature of pyroptotic cell death, results in organelle damage and leakage of intracellular contents into the extracellular space. **C** The cleavage of GSMDE and releasing HMGB1 from cytosol to extracellular space in PAMT-001-treated HT-29 cells.

1. **Supplementary table (patient list)**

|  | **LD50(uM) PAMT-001** | **Sex** | **Age** | **BM blast (%)** | **Risk group** | **Complete remission** | **Status** |
| --- | --- | --- | --- | --- | --- | --- | --- |
| #111 | 4.592 | F | 28 | 78.5 | Poor | No | n/a |
| #112 | 15.02 | F | 80 | 30.9 | Poor | Yes | Alive |
| #115 | 2.732 | F | 23 | 91.3 | Favorable | Yes | Alive |
| #121 | 2.461 | F | 23 | 65.1 | Intermediate | Yes | Death |
| #99 | 2.655 | M | 65 | 68.4 | Favorable | No | Alive |
| #129 | 2.619 | F | 73 | 2.4 | Intermediate | Yes | Alive |
| #131 | 2.201 | F | 25 | 25.3 | Intermediate | Yes | Alive |
| #132 | 4.898 | F | 73 | 60.7 | Poor | No | Alive |
| #135 | 3.17 | M | 62 | 74.3 | Poor | No | Death |
| #138 | 4.634 | M | 53 | 48.5 | Poor | Yes | Alive |
| #140 | 1.211 | F | 81 | 77.6 | Poor | No | n/a |
| #141 | 5.517 | F | 29 | 79 | Poor | No | Death |
| #144 | 1.517 | M | 68 | 51.4 | Intermediate | Yes | Alive |
| #146 | 1.889 | F | 74 | 57.9 | Favorable | Yes | Alive |
| #120 | 2.621 | F | 45 | 50 | Poor | Yes | Alive |
| #117 | 3.029 | M | 82 | 76.6 | Intermediate | No | Death |
| #156 | 3.023 | M | 70 | 72.7 | Favorable | No | Death |
| #142 | 2.435 | M | 78 | 46.8 | Intermediate | Yes | Alive |
| #108 | 1.181 | M | 68 | 68.2 | Intermediate | Yes | Alive |
| #165 | 2.375 | M | 68 | 77.2 | Intermediate | No | Death |
| #100 | 1.608 | F | 72 | 74.4 | Intermediate | Yes | Alive |
| #64 | 4.755 | M | 73 | 38.1 | Favorable | No | Death |
| #71 | 2.962 | M | 54 | 53.1 | Poor | No | Death |
| #104 | 1.626 | F | 58 | 92.2 | Favorable | Yes | Alive |
| #57 | 4.759 | M | 68 | 28.7 | Intermediate | Yes | Death |
| #167 | 3.106 | F | 62 | 58.2 | Poor | No | Alive |
| #168 | 3.567 | F | 67 | 78 | Favorable | Yes | Alive |
| #169 | 4.444 | M | 70 | 27.2 | Poor | No | Alive |

**REFERENCES**

1. Quentmeier H, Zaborski M, Drexler HG. The human bladder carcinoma cell line 5637 constitutively secretes functional cytokines. Leuk Res. 1997;21(4):343-50.

2. Niu X, Wang G, Wang Y, Caldwell JT, Edwards H, Xie C, et al. Acute myeloid leukemia cells harboring MLL fusion genes or with the acute promyelocytic leukemia phenotype are sensitive to the Bcl-2-selective inhibitor ABT-199. Leukemia. 2014;28(7):1557-60.

3. Qiao X, Ma J, Knight T, Su Y, Edwards H, Polin L, et al. The combination of CUDC-907 and gilteritinib shows promising in vitro and in vivo antileukemic activity against FLT3-ITD AML. Blood Cancer J. 2021;11(6):111.

4. Jurrus E, Engel D, Star K, Monson K, Brandi J, Felberg LE, et al. Improvements to the APBS biomolecular solvation software suite. Protein Sci. 2018;27(1):112-28.

5. Word JM, Lovell SC, Richardson JS, Richardson DC. Asparagine and glutamine: using hydrogen atom contacts in the choice of side-chain amide orientation. J Mol Biol. 1999;285(4):1735-47.

6. O'Boyle NM, Banck M, James CA, Morley C, Vandermeersch T, Hutchison GR. Open Babel: An open chemical toolbox. J Cheminform. 2011;3:33.

7. Kochnev Y, Durrant JD. FPocketWeb: protein pocket hunting in a web browser. J Cheminform. 2022;14(1):58.

8. Volkamer A, Kuhn D, Rippmann F, Rarey M. DoGSiteScorer: a web server for automatic binding site prediction, analysis and druggability assessment. Bioinformatics. 2012;28(15):2074-5.

9. Eberhardt J, Santos-Martins D, Tillack AF, Forli S. AutoDock Vina 1.2.0: New Docking Methods, Expanded Force Field, and Python Bindings. J Chem Inf Model. 2021;61(8):3891-8.

10. Kochnev Y, Hellemann E, Cassidy KC, Durrant JD. Webina: an open-source library and web app that runs AutoDock Vina entirely in the web browser. Bioinformatics. 2020;36(16):4513-5.

11. Kochnev Y, Ahmed M, Maldonado AM, Durrant JD. MolModa: accessible and secure molecular docking in a web browser. Nucleic Acids Res. 2024;52(W1):W498-W506.

12. Yang Y, Zhang G, Yang T, Gan J, Xu L, Yang H. A flow-cytometry-based protocol for detection of mitochondrial ROS production under hypoxia. STAR Protoc. 2021;2(2):100466.

13. Chen X, Glytsou C, Zhou H, Narang S, Reyna DE, Lopez A, et al. Targeting Mitochondrial Structure Sensitizes Acute Myeloid Leukemia to Venetoclax Treatment. Cancer Discov. 2019;9(7):890-909.
