# Supplementary material for: A novel PAAoptosis-inducing ERRα-targeting compound for combating hematopoietic and solid cancers
Source: Cell Death Discov. 2026 Mar 26;12:188. doi: 10.1038/s41420-026-03010-4 (PMC13139410; doi:10.1038/s41420-026-03010-4)
Supplement: Supplementary file 2 — Full and uncropped western blots [file 41420_2026_3010_MOESM2_ESM.pdf]

Fig. 2G

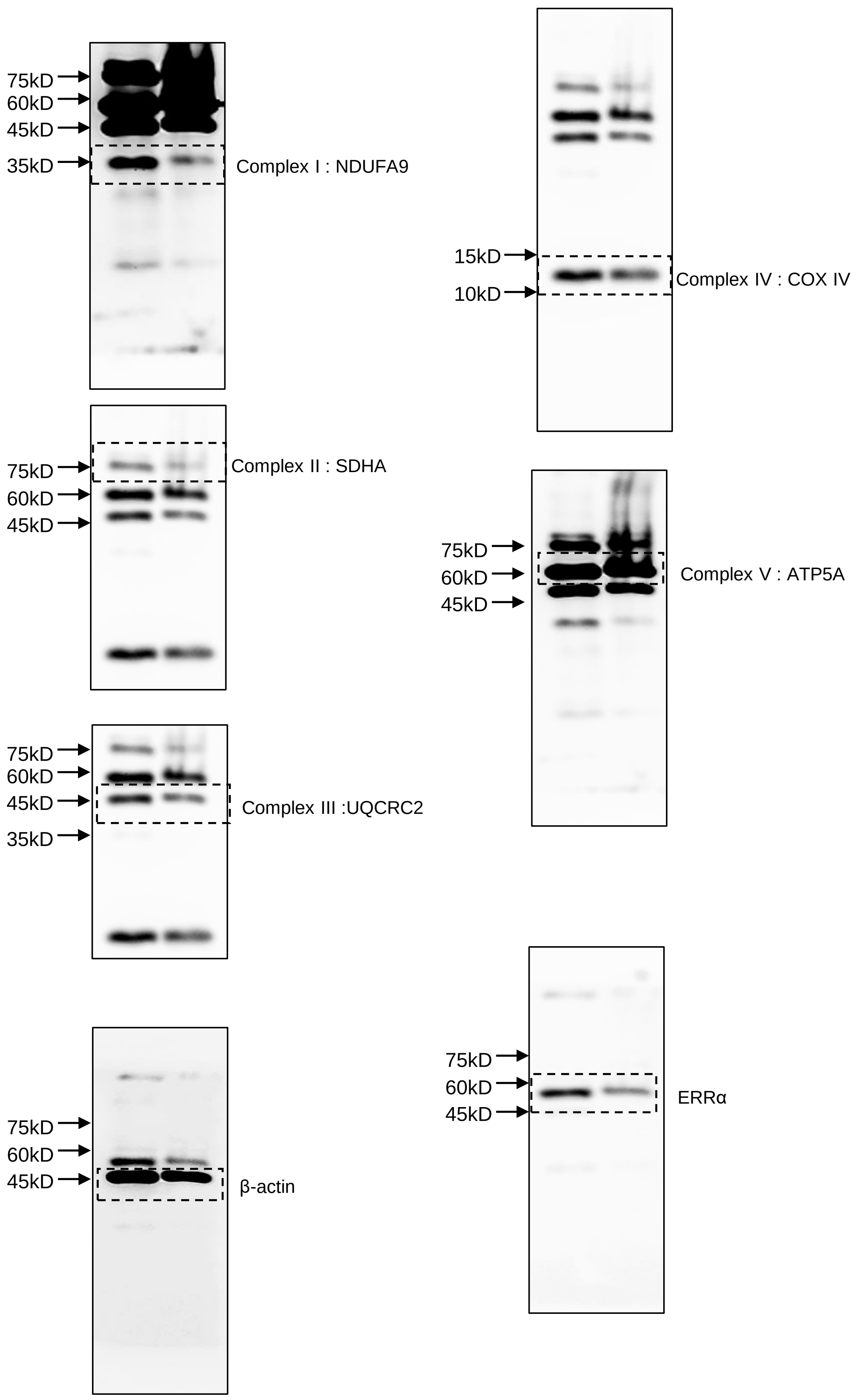
75kD
60kD
45kD
35kD
Complex I : NDUFA9
75kD
60kD
45kD
Complex II : SDHA
75kD
60kD
45kD
35kD
Complex III :UQCRC2
75kD
60kD
45kD
β-actin
15kD
10kD
Complex IV : COX IV
75kD
60kD
45kD
Complex V : ATP5A
75kD
60kD
45kD
ERRα

SFig. 2A,B

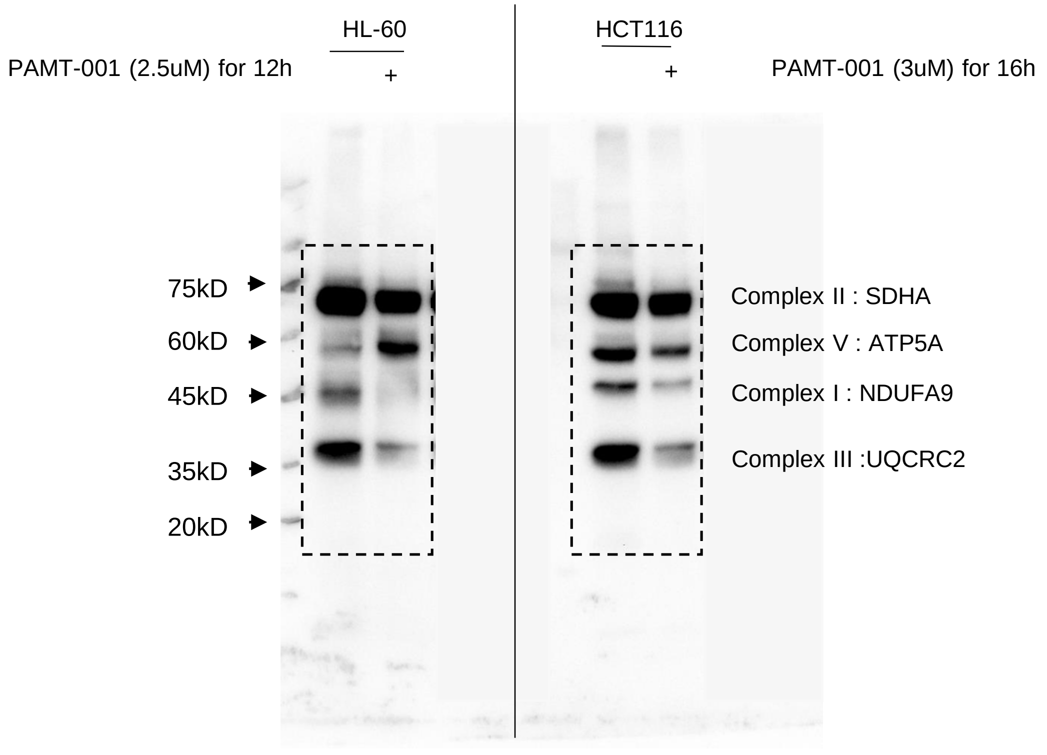

HL-60
PAMT-001 (2.5uM) for 12h
+
HCT116
+
PAMT-001 (3uM) for 16h
75kD
60kD
45kD
35kD
20kD
Complex II : SDHA
Complex V : ATP5A
Complex I : NDUFA9
Complex III :UQCRC2

SFig. 2C

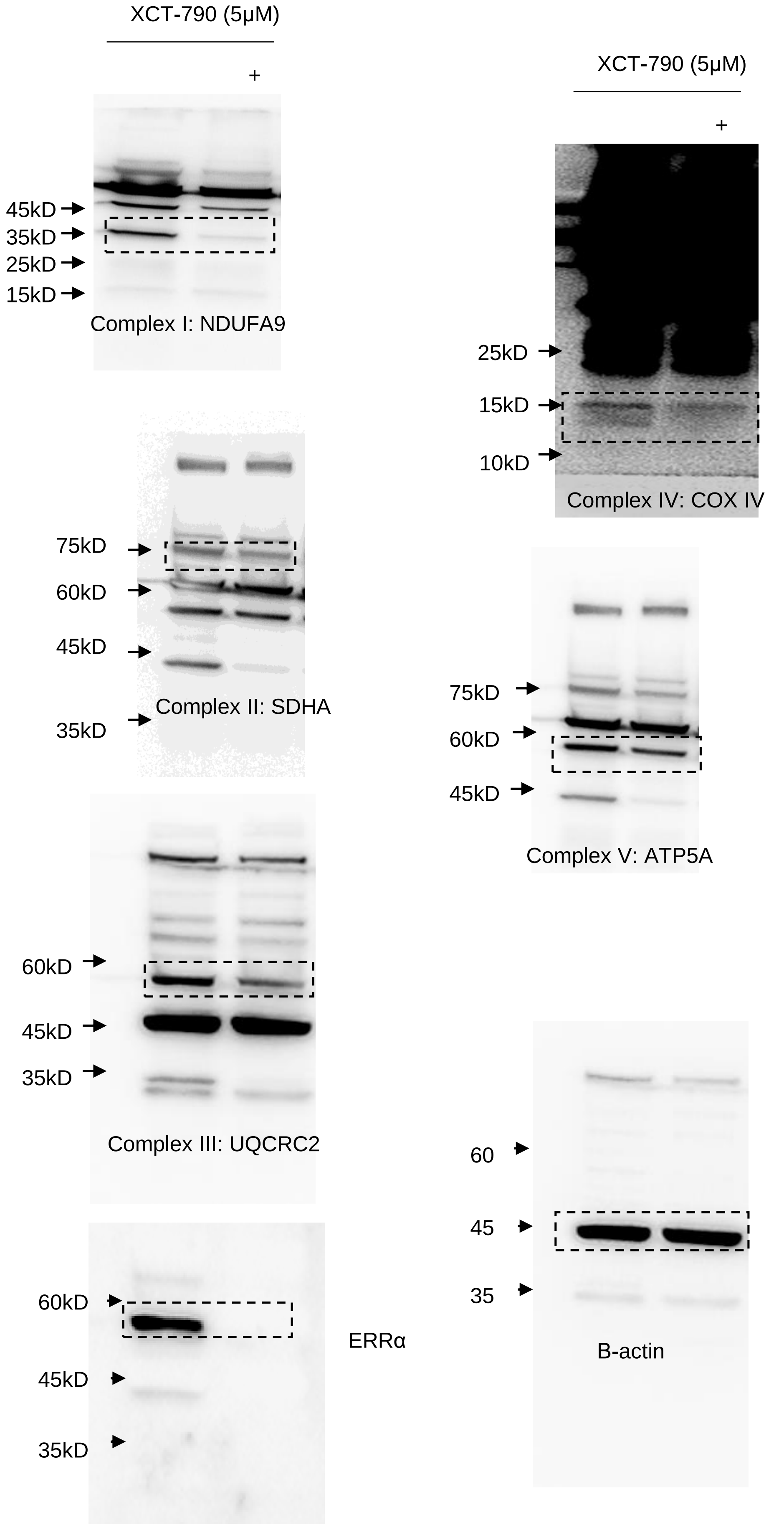
XCT-790 (5μM)
+
45kD
35kD
25kD
15kD
Complex I: NDUFA9
75kD
60kD
45kD
Complex II: SDHA
35kD
60kD
45kD
35kD
Complex III: UQCRC2
60kD
45kD
35kD
ERRα
XCT-790 (5μM)
+
25kD
15kD
10kD
Complex IV: COX IV
75kD
60kD
45kD
Complex V: ATP5A
60
45
35
B-actin

Fig. 2F

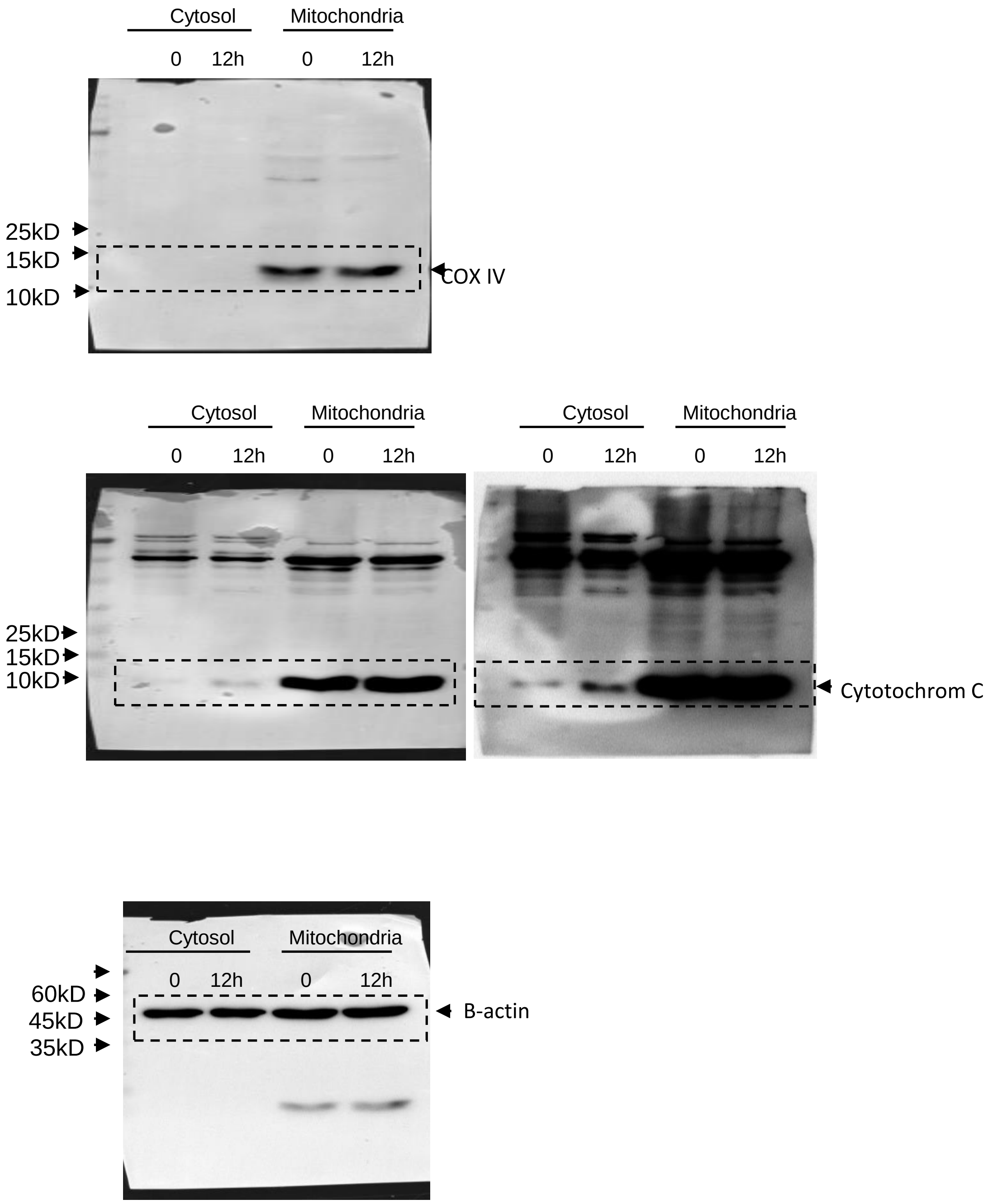

Cytosol
Mitochondria
0
12h
0
12h
25kD
15kD
10kD
COX IV
Cytosol
Mitochondria
0
12h
0
12h
Cytosol
Mitochondria
0
12h
0
12h
25kD
15kD
10kD
Cytotochrom C
Cytosol
Mitochondria
0
12h
0
12h
60kD
45kD
35kD
B-actin

SFig. 3C

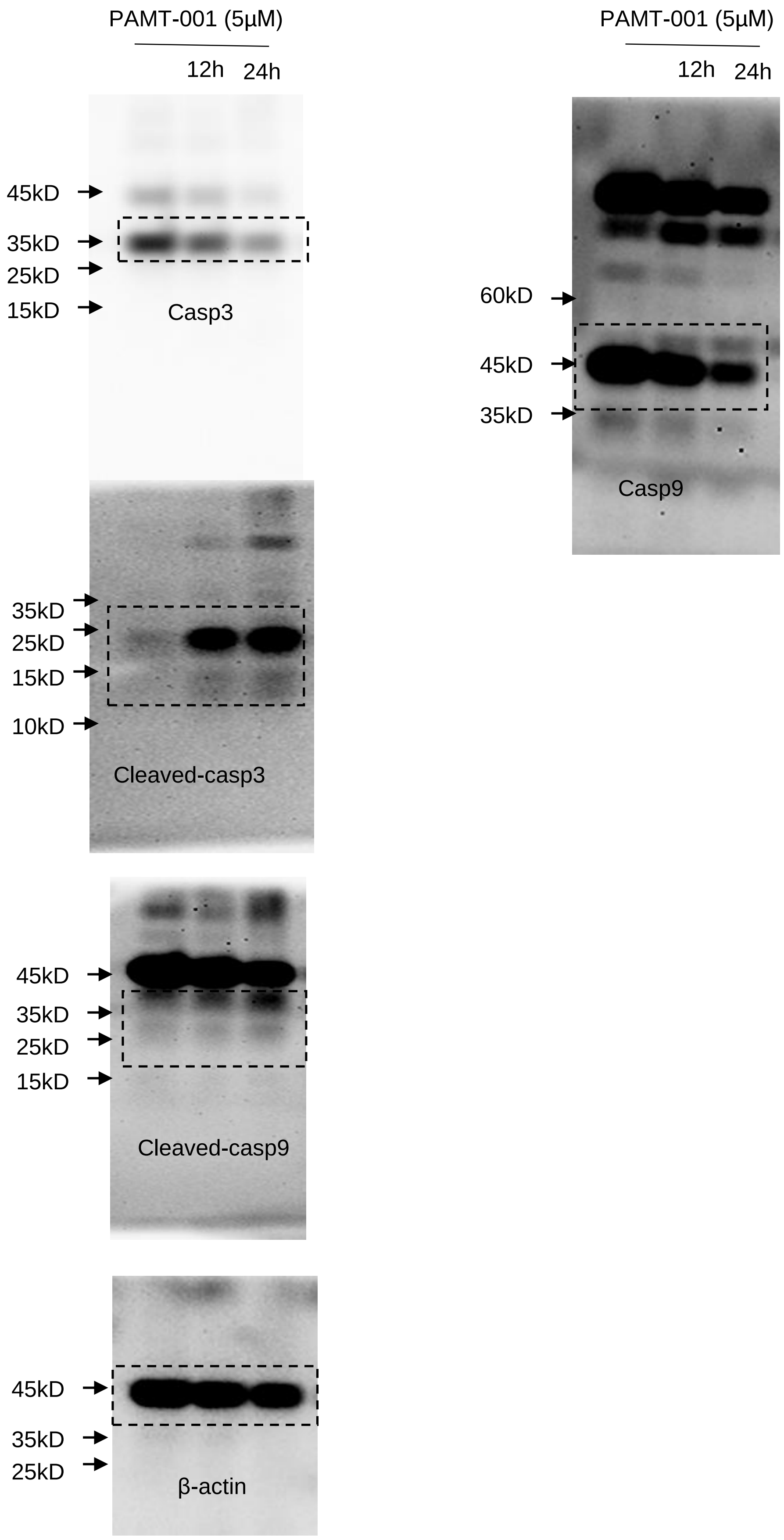
PAMT-001 (5µM)
12h
24h
45kD
35kD
25kD
15kD
Casp3
35kD
25kD
15kD
10kD
Cleaved-casp3
45kD
35kD
25kD
15kD
Cleaved-casp9
45kD
35kD
25kD
β-actin
PAMT-001 (5µM)
12h
24h
60kD
45kD
35kD
Casp9

Fig. 3E

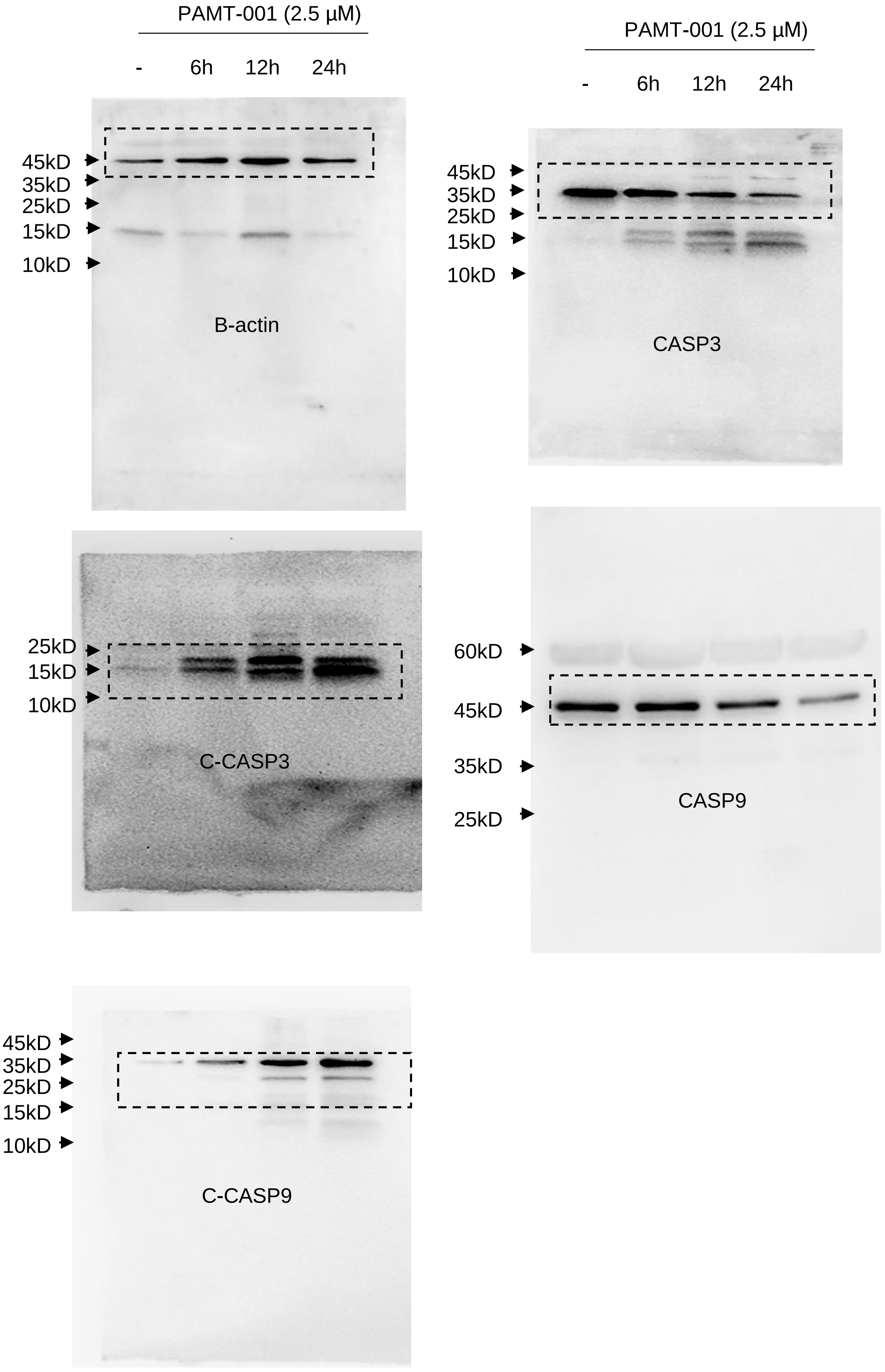

PAMT-001 (2.5 µM)
- 6h 12h 24h
45kD
35kD
25kD
15kD
10kD
B-actin
PAMT-001 (2.5 µM)
- 6h 12h 24h
45kD
35kD
25kD
15kD
10kD
CASP3
25kD
15kD
10kD
C-CASP3
60kD
45kD
35kD
25kD
CASP9
45kD
35kD
25kD
15kD
10kD
C-CASP9

Fig. 3E

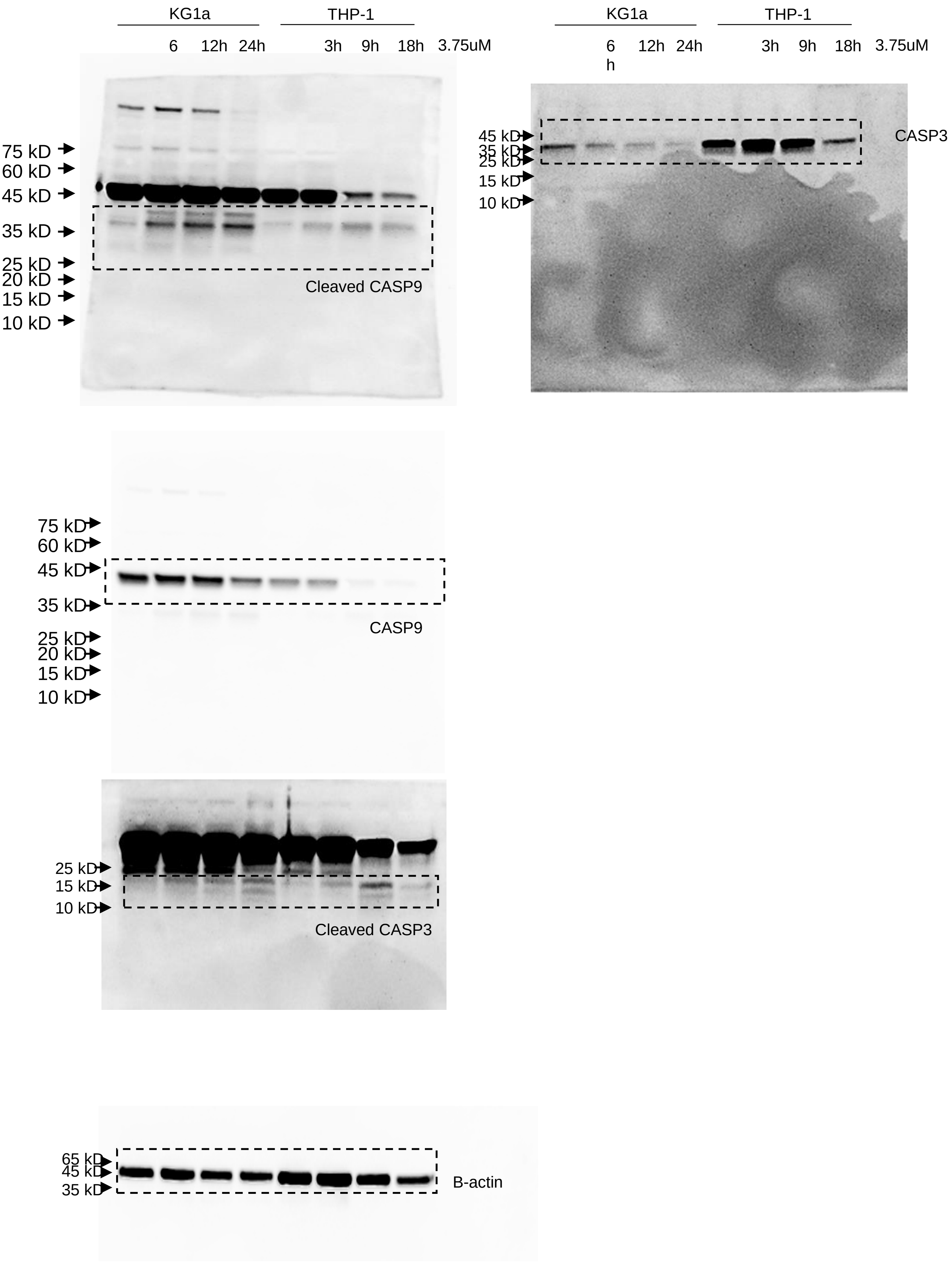

KG1a
THP-1
6
12h
24h
3h
9h
18h
3.75uM
75 kD
60 kD
45 kD
35 kD
25 kD
20 kD
15 kD
10 kD
Cleaved CASP9
KG1a
THP-1
6
h
12h
24h
3h
9h
18h
3.75uM
45 kD
35 kD
25 kD
15 kD
10 kD
CASP3
75 kD
60 kD
45 kD
35 kD
25 kD
20 kD
15 kD
10 kD
CASP9
25 kD
15 kD
10 kD
Cleaved CASP3
65 kD
45 kD
35 kD
B-actin

Fig. 4F, G

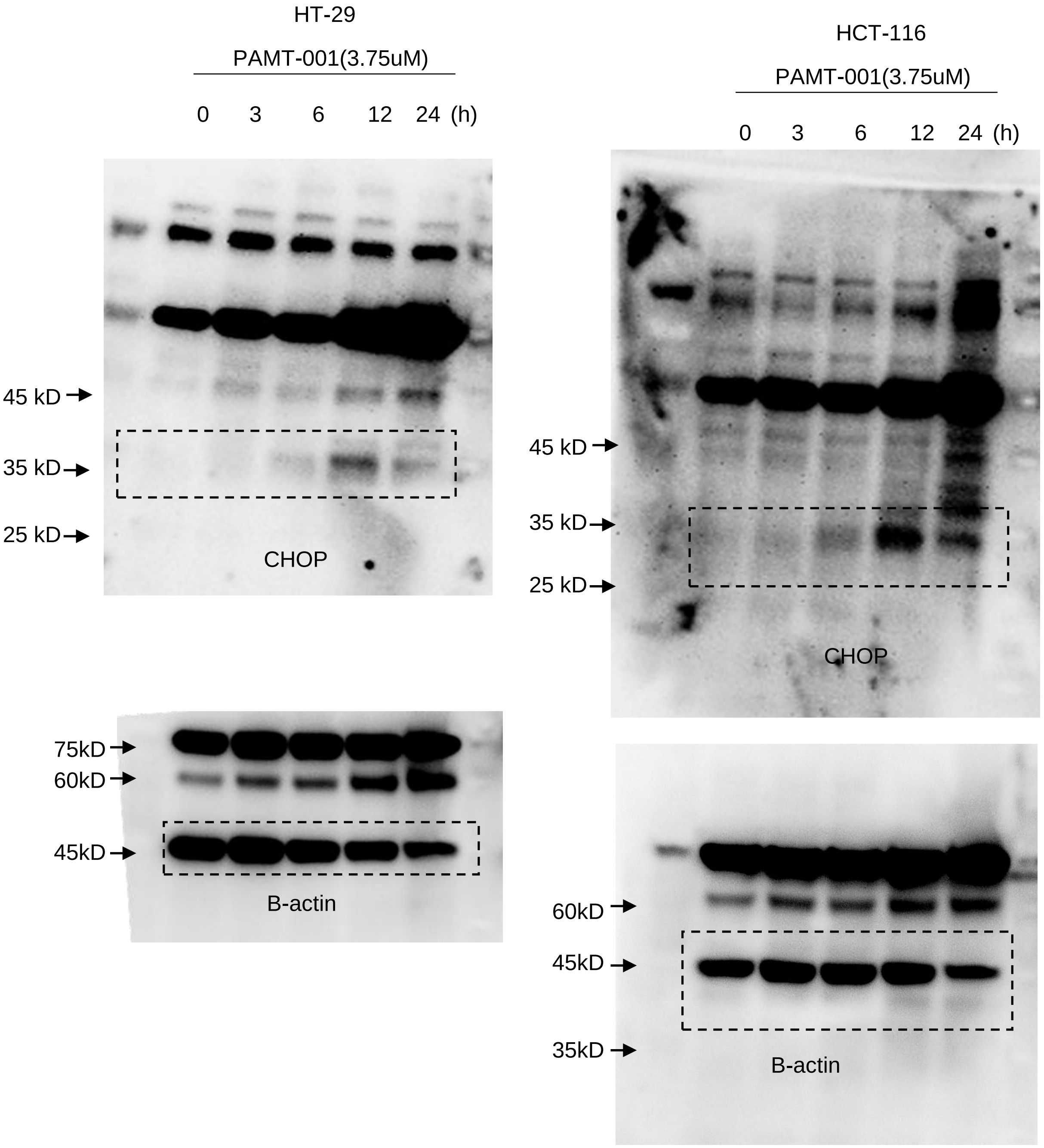
HT-29
PAMT-001(3.75uM)
0 3 6 12 24 (h)
45 kD
35 kD
25 kD
CHOP
75kD
60kD
45kD
B-actin
HCT-116
PAMT-001(3.75uM)
0 3 6 12 24 (h)
45 kD
35 kD
25 kD
CHOP
60kD
45kD
35kD
B-actin

SFig. 4A

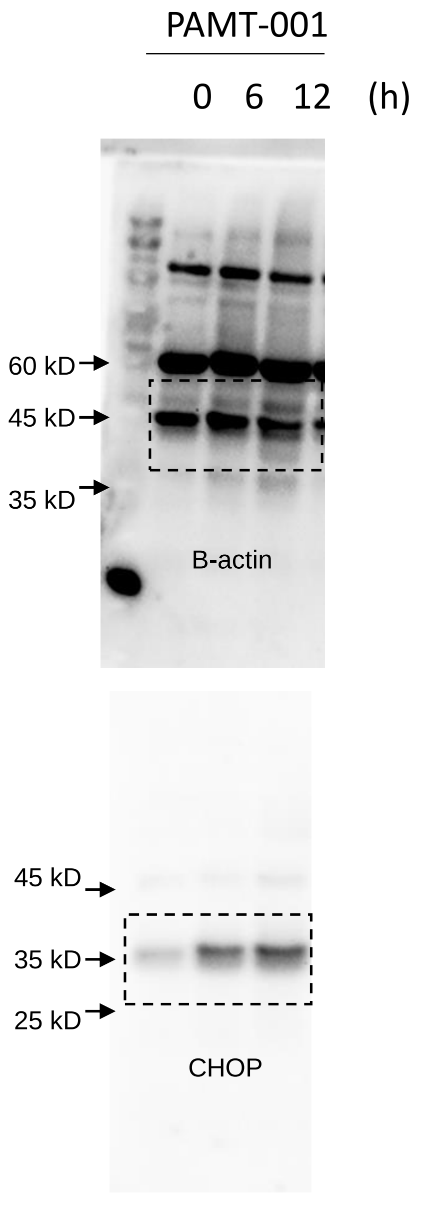

SFig. 4B

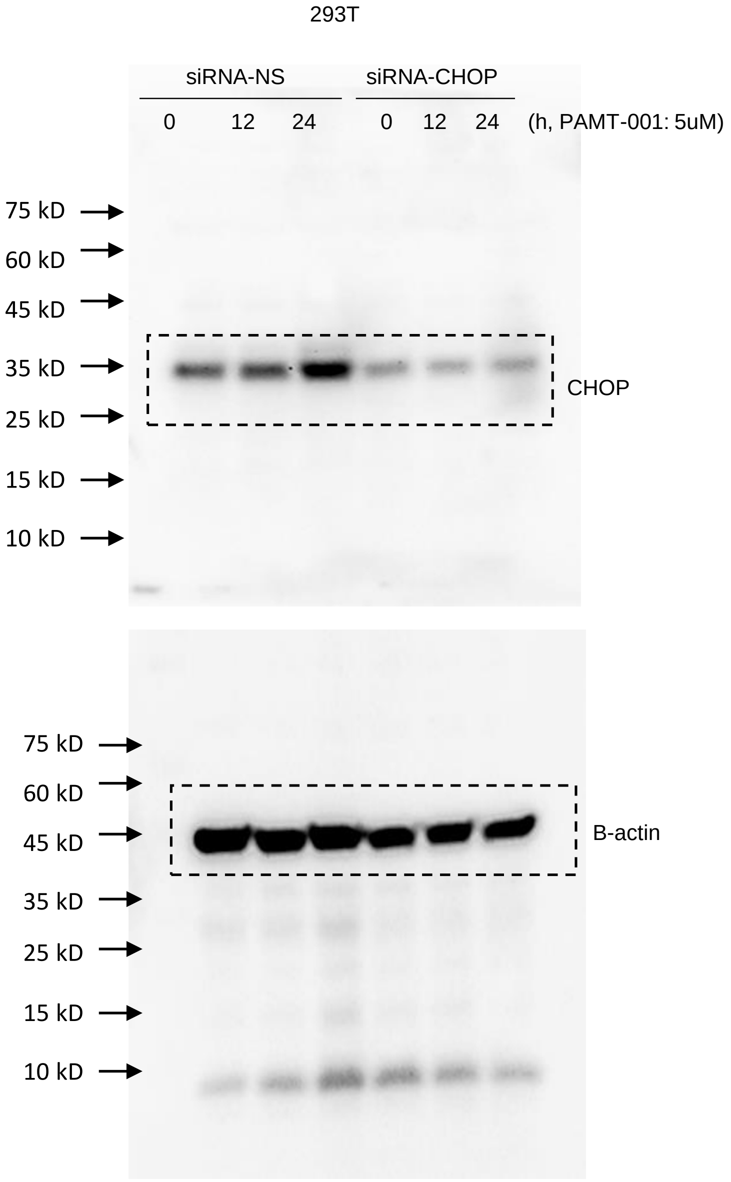
293T
siRNA-NS
siRNA-CHOP
0
12
24
0
12
24
(h, PAMT-001: 5uM)
75 kD
60 kD
45 kD
35 kD
25 kD
15 kD
10 kD
CHOP
75 kD
60 kD
45 kD
35 kD
25 kD
15 kD
10 kD
B-actin

Fig. 5A

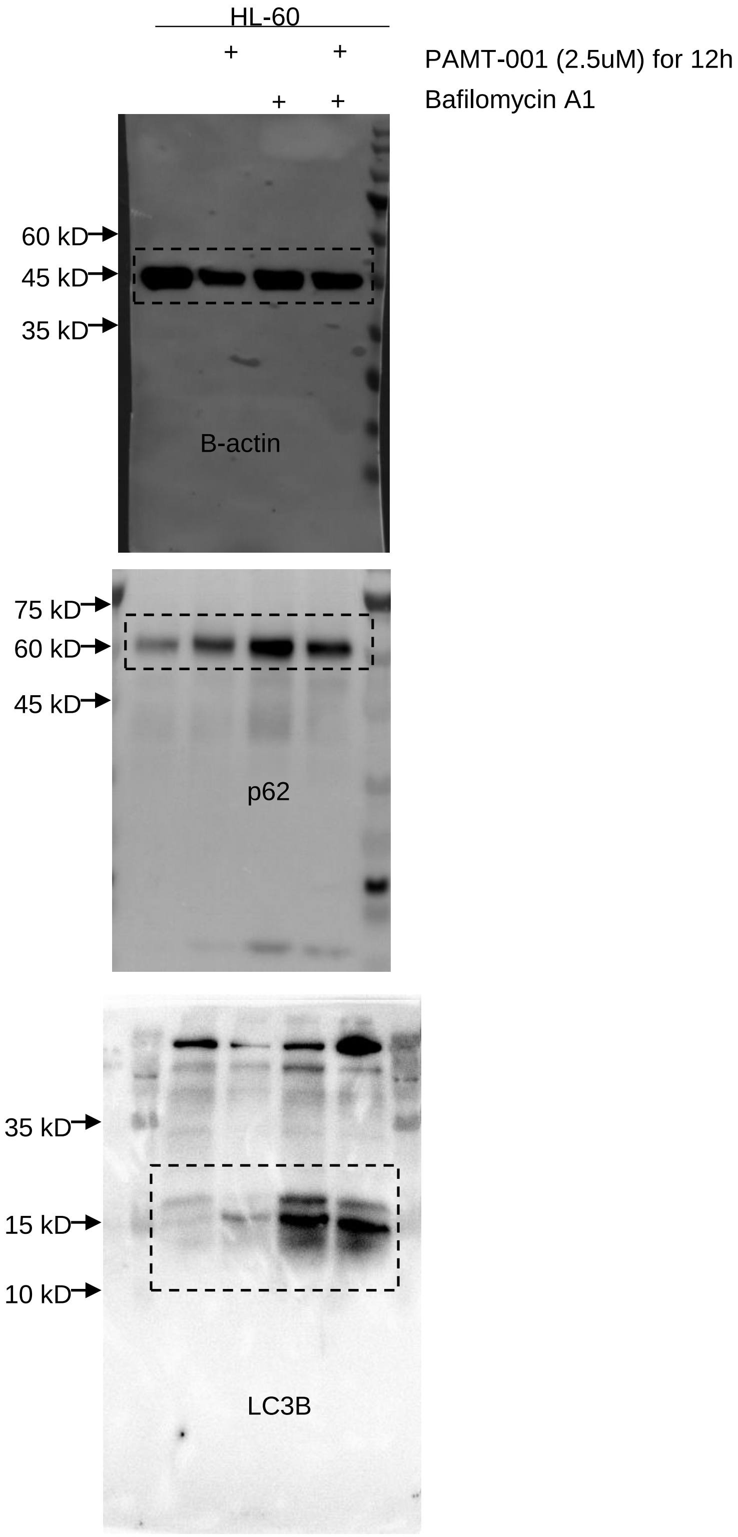

HL-60
+
+
PAMT-001 (2.5uM) for 12h
+
+
Bafilomycin A1
60 kD
45 kD
35 kD
B-actin
75 kD
60 kD
45 kD
p62
35 kD
15 kD
10 kD
LC3B

Fig. 5A

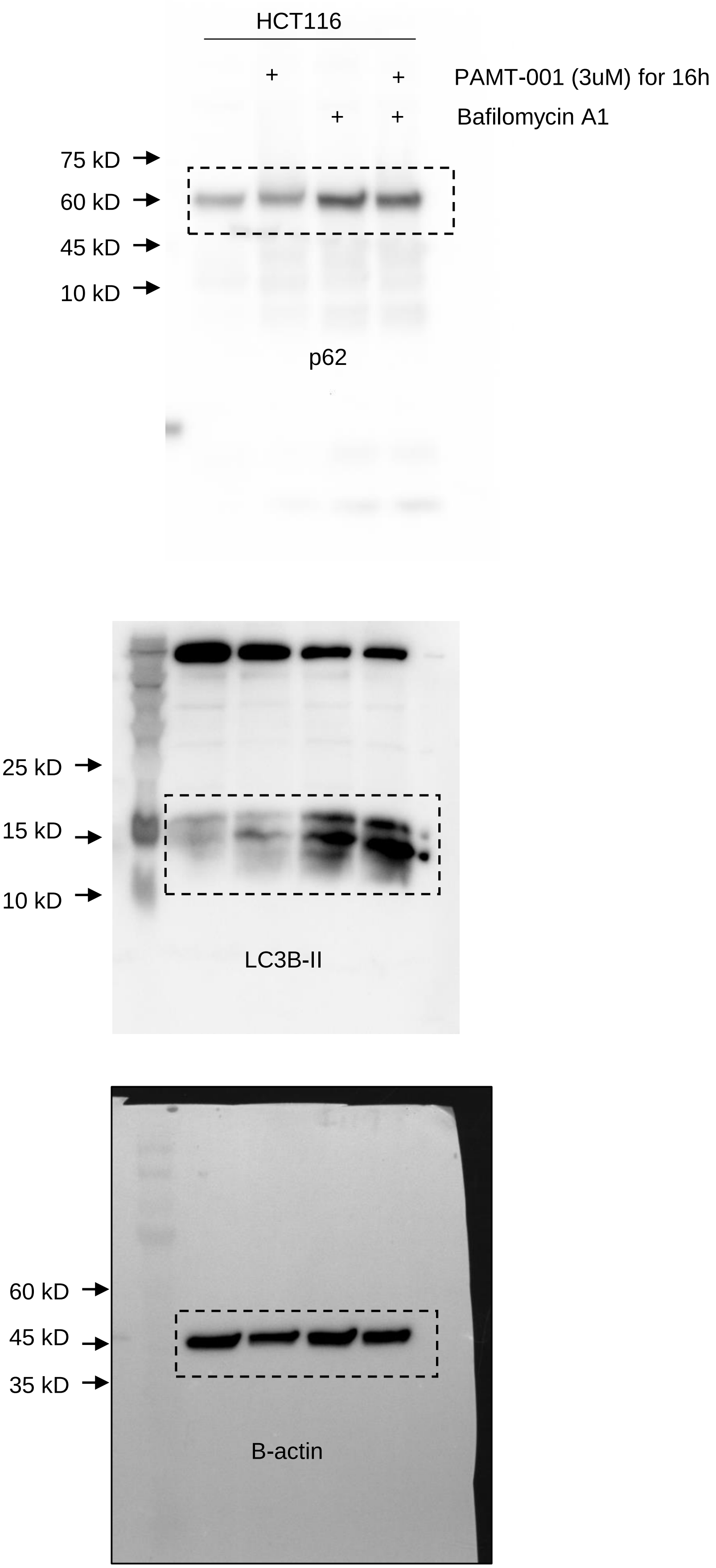
HCT116
+ + PAMT-001 (3uM) for 16h
+ + Bafilomycin A1
75 kD
60 kD
45 kD
10 kD
p62
25 kD
15 kD
10 kD
LC3B-II
60 kD
45 kD
35 kD
B-actin

Fig. 5F

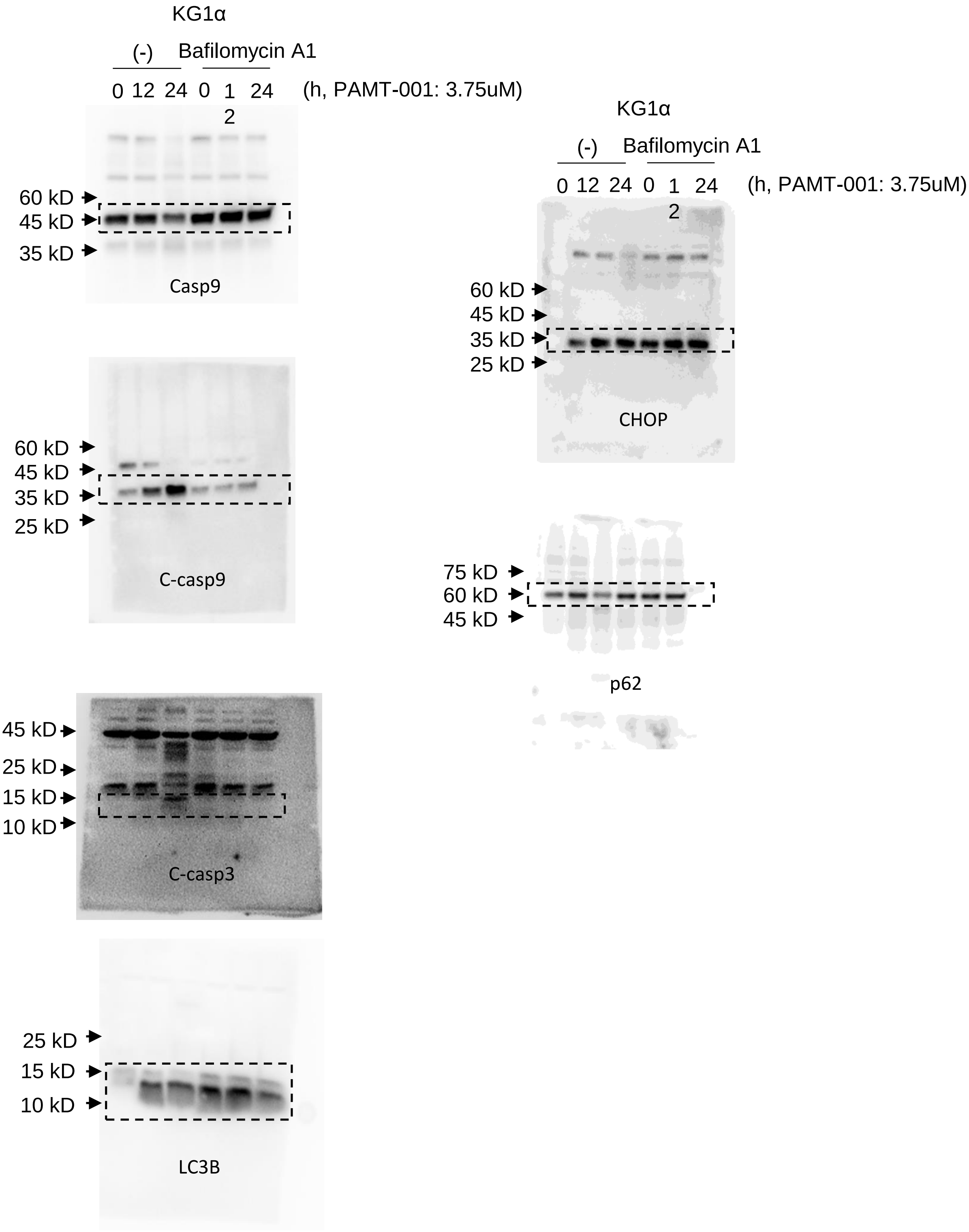
KG1α
(-) Bafilomycin A1
0 12 24 0 12 24 (h, PAMT-001: 3.75uM)
60 kD
45 kD
35 kD
Casp9
60 kD
45 kD
35 kD
25 kD
C-casp9
45 kD
25 kD
15 kD
10 kD
C-casp3
25 kD
15 kD
10 kD
LC3B
KG1α
(-) Bafilomycin A1
0 12 24 0 12 24 (h, PAMT-001: 3.75uM)
60 kD
45 kD
35 kD
25 kD
CHOP
75 kD
60 kD
45 kD
p62

Fig. 5F and SFig. 5B

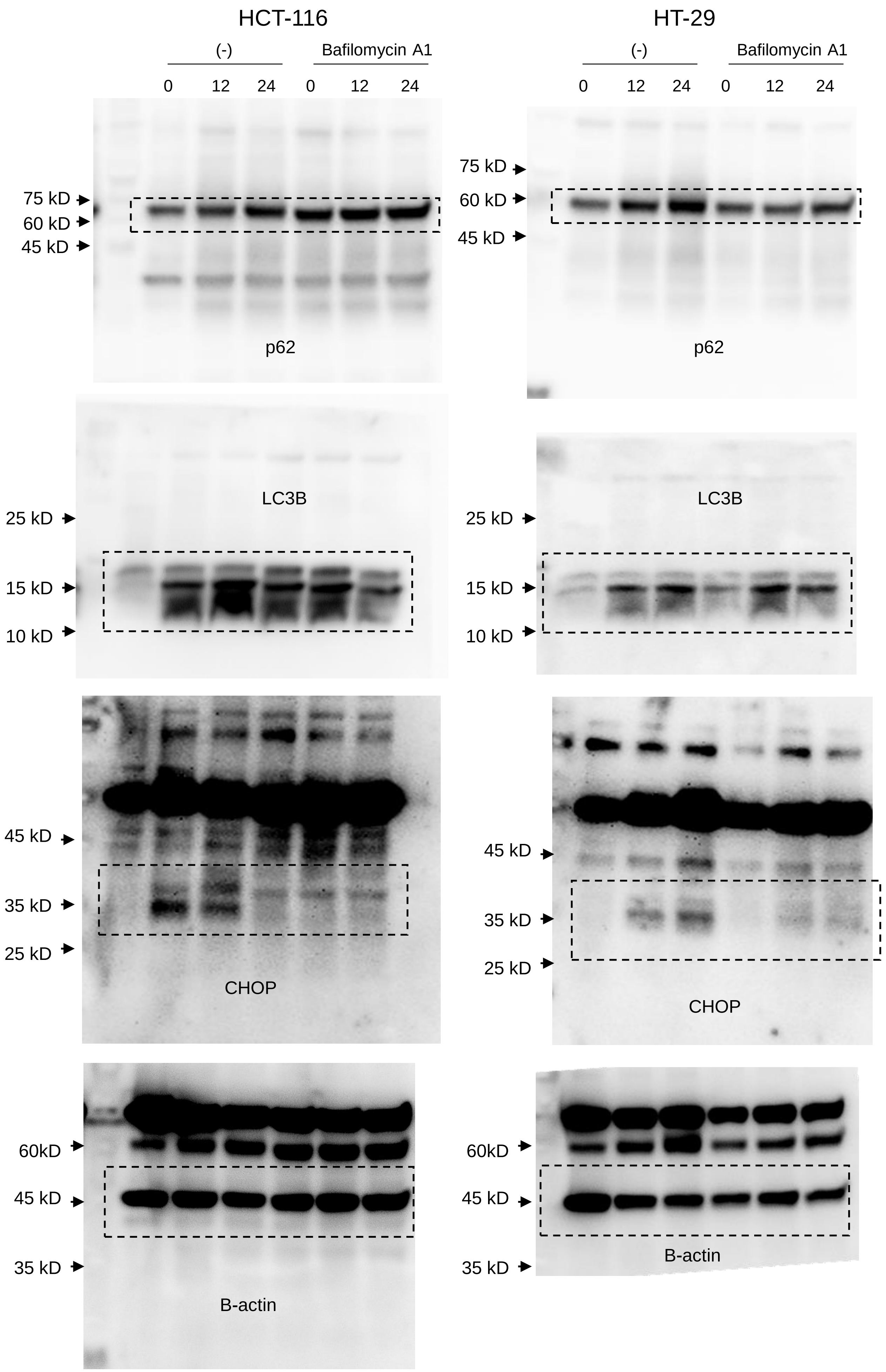

Fig. 5F and SFig. 5B

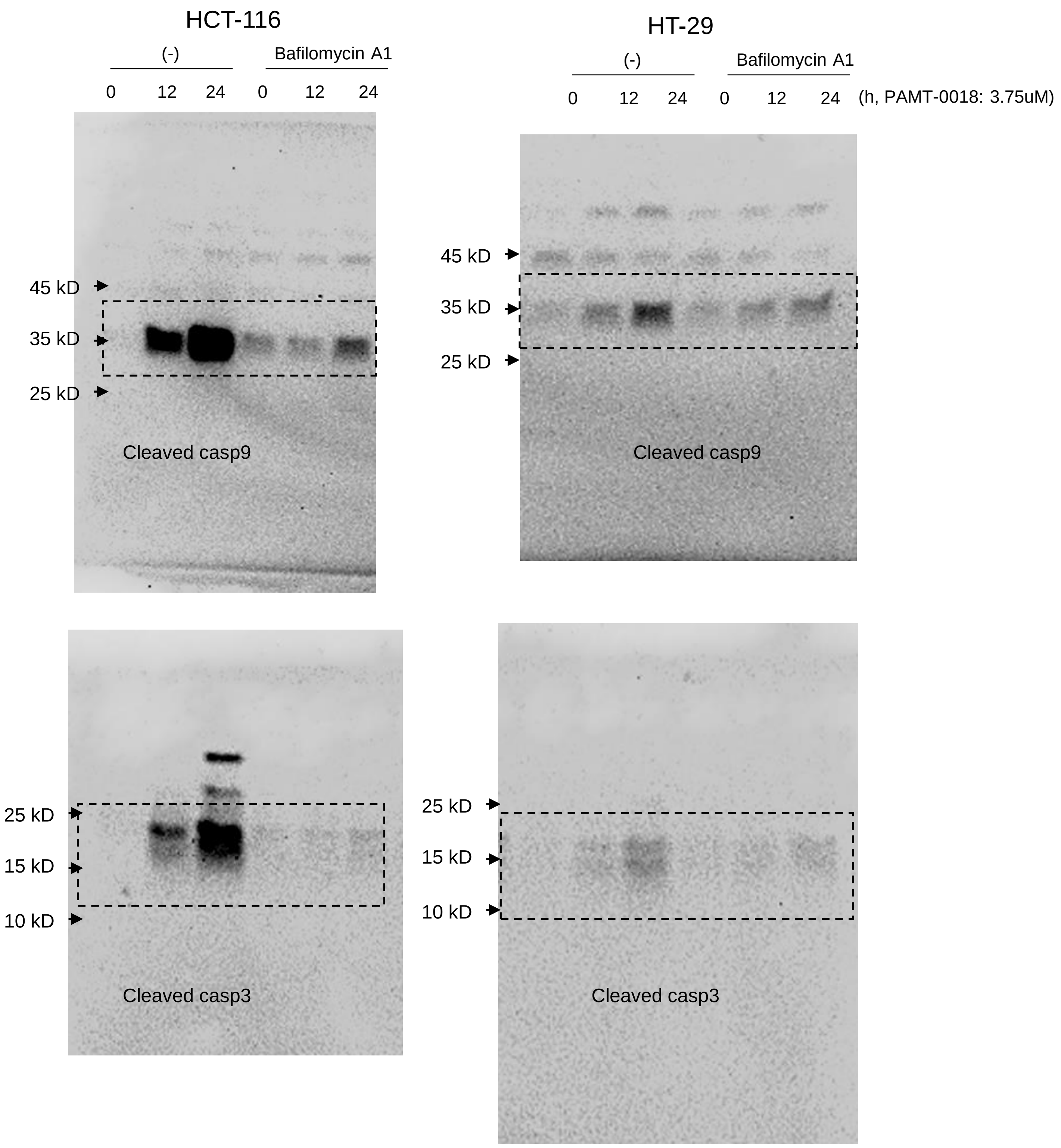

SFig. 5A

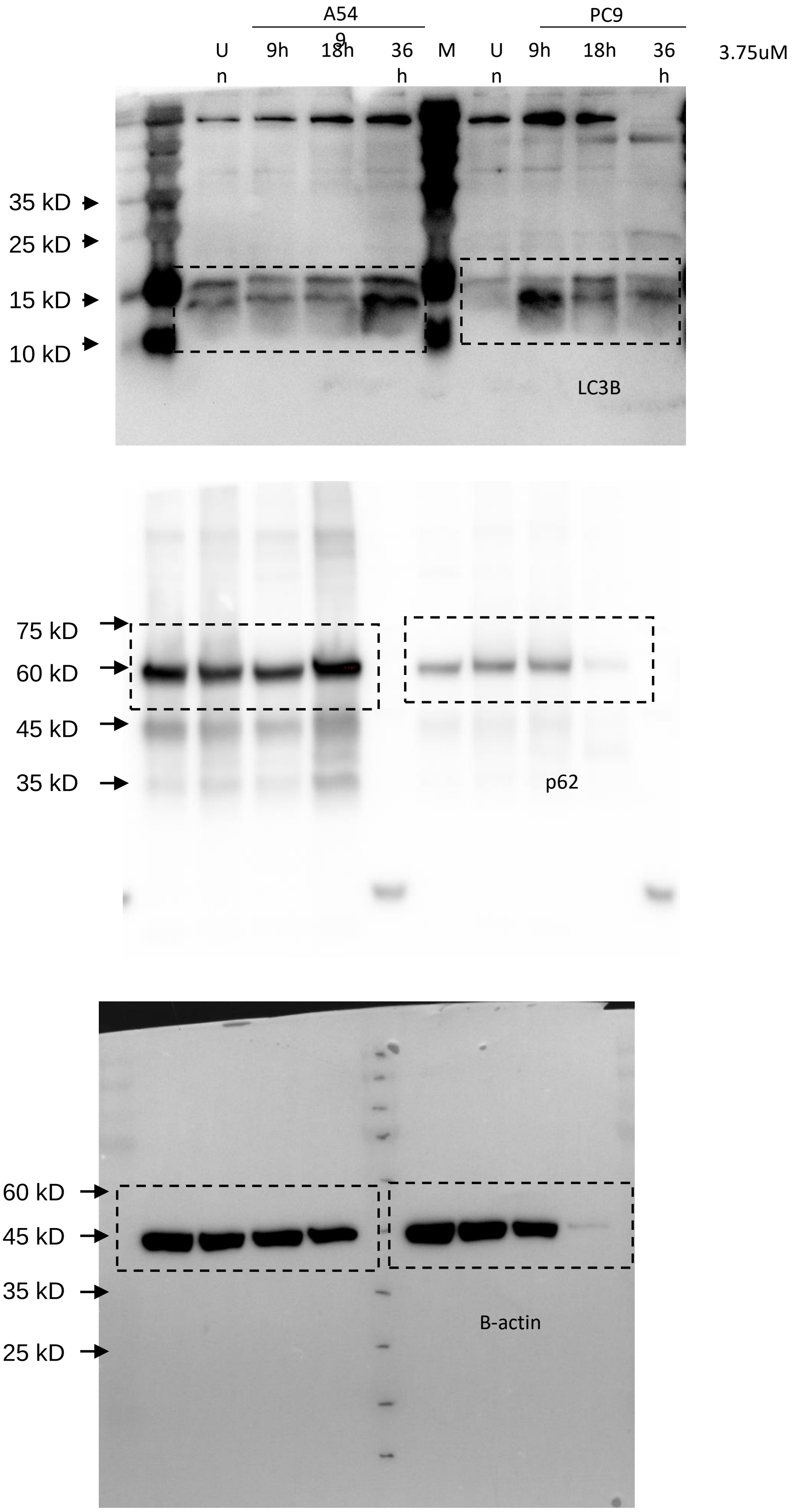
A54
9
PC9
U
n
9h
18h
36
h
M
U
n
9h
18h
36
h
3.75uM
35 kD
25 kD
15 kD
10 kD
LC3B
75 kD
60 kD
45 kD
35 kD
p62
60 kD
45 kD
35 kD
25 kD
B-actin

SFig. 6B and Fig. 6D

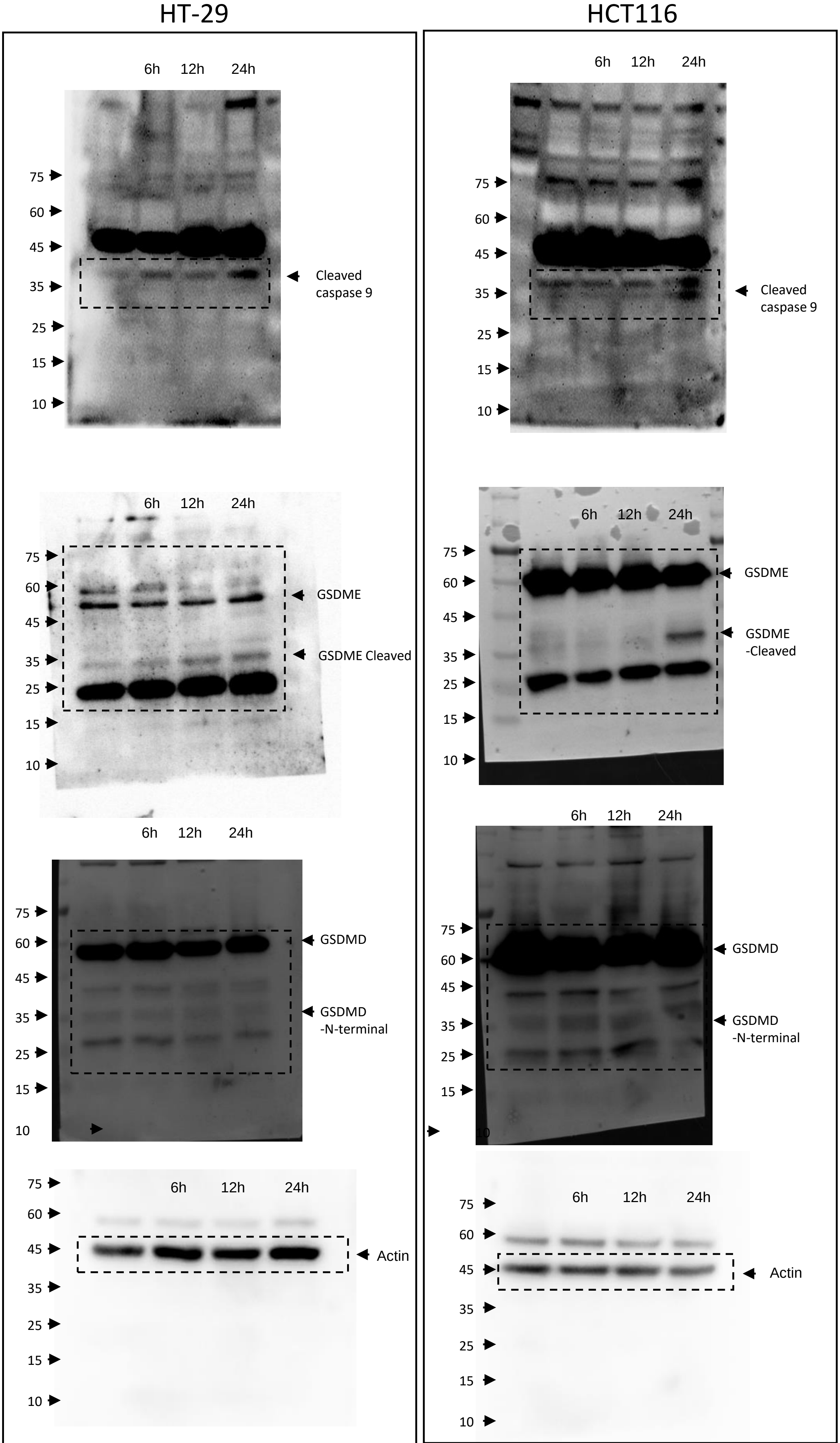

SFig. 6B and Fig. 6D

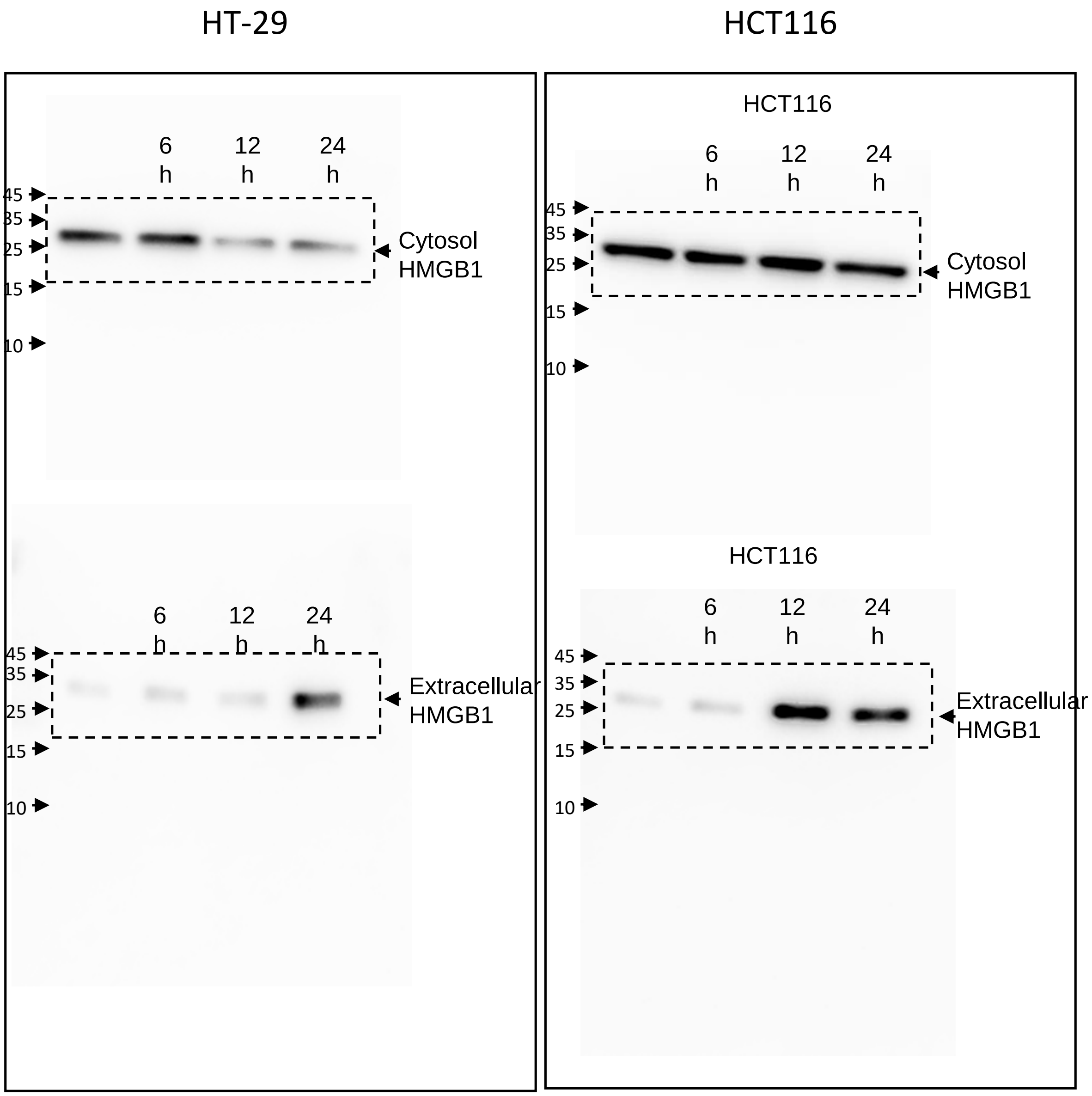

Fig. 6D

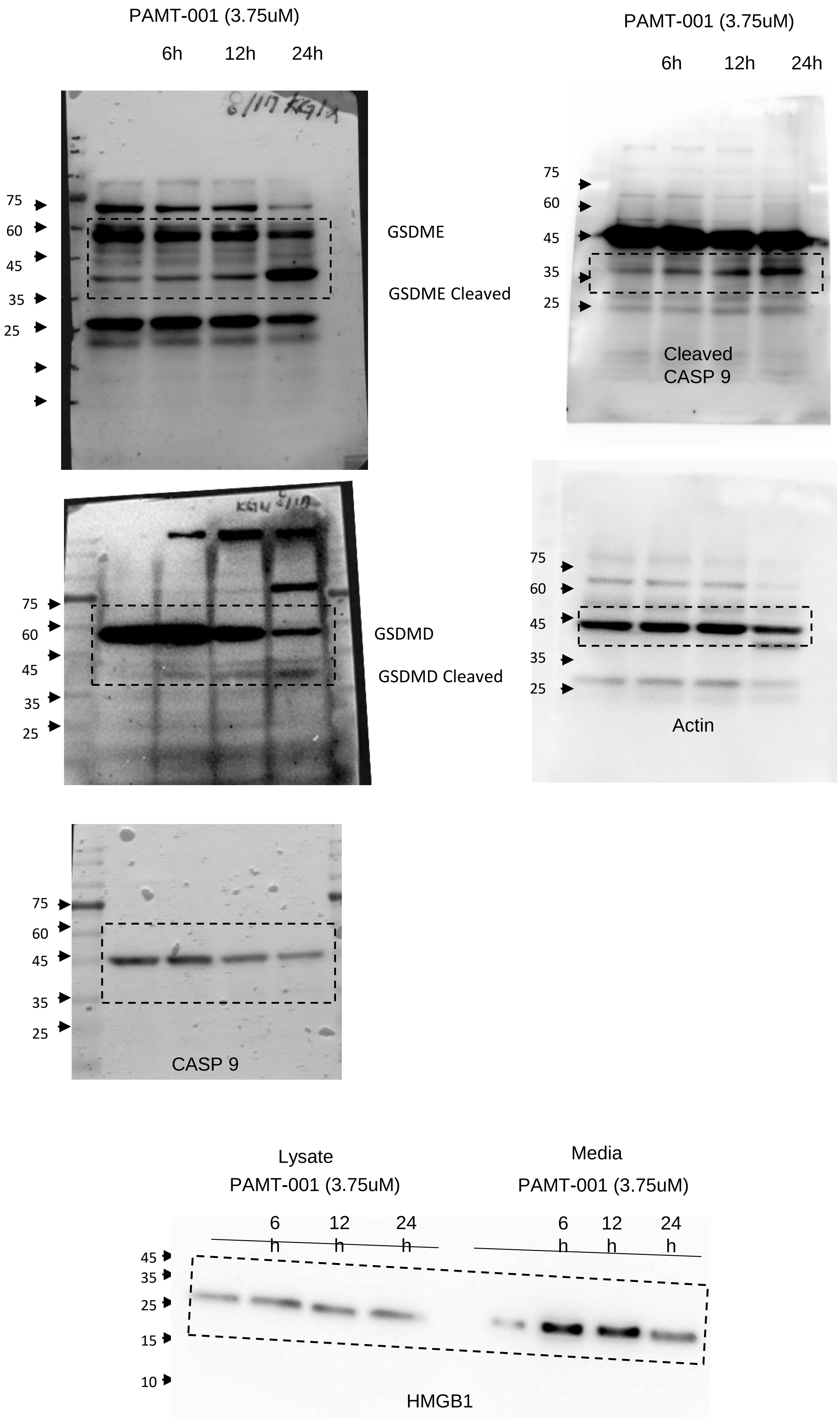
PAMT-001 (3.75uM)
6h
12h
24h
75
60
45
35
25
GSDME
GSDME Cleaved
PAMT-001 (3.75uM)
6h
12h
24h
75
60
45
35
25
Cleaved
CASP 9
75
60
45
35
25
GSDMD
GSDMD Cleaved
75
60
45
35
25
Actin
75
60
45
35
25
CASP 9
Lysate
PAMT-001 (3.75uM)
Media
PAMT-001 (3.75uM)
6
h
12
h
24
h
6
h
12
h
24
h
45
35
25
15
10
HMGB1
